# Differentiation of Spiral Ganglion Neurons from Human Dental Pulp Stem Cells: A Further Step towards Autologous Auditory Nerve Recovery

**DOI:** 10.3390/ijms25169115

**Published:** 2024-08-22

**Authors:** Yassine Messat, Marta Martin-Fernandez, Said Assou, Keshi Chung, Frederic Guérin, Csilla Gergely, Frederic Cuisinier, Azel Zine

**Affiliations:** 1 LBN, Laboratory of Bioengineering and Nanoscience, University of Montpellier, 34193 Montpellier, France; 2 L2C, Laboratoire Charles Coulomb, University of Montpellier, CNRS, 34095 Montpellier, France; 3 IRMB, Institute for Regenerative Medicine & Biotherapy, University of Montpellier, INSERM, CHU Montpellier, 34295 Montpellier, France; said.assou@inserm.fr; 4 Faculté de Médecine, University of Montpellier, 34090 Montpellier, France

**Keywords:** adult dental pulp stem cells, differentiation, human otic neural progenitors, spiral ganglion neurons, mechanical properties, cell therapy

## Abstract

The degeneration of spiral ganglion neurons (SGNs), which convey auditory signals from hair cells to the brain, can be a primary cause of sensorineural hearing loss (SNHL) or can occur secondary to hair cell loss. Emerging therapies for SNHL include the replacement of damaged SGNs using stem cell-derived otic neuronal progenitors (ONPs). However, the availability of renewable, accessible, and patient-matched sources of human stem cells is a prerequisite for successful replacement of the auditory nerve. In this study, we derived ONP and SGN-like cells by a reliable and reproducible stepwise guidance differentiation procedure of self-renewing human dental pulp stem cells (hDPSCs). This in vitro differentiation protocol relies on the modulation of BMP and TGFβ pathways using a free-floating 3D neurosphere method, followed by differentiation on a Geltrex-coated surface using two culture paradigms to modulate the major factors and pathways involved in early otic neurogenesis. Gene and protein expression analyses revealed efficient induction of a comprehensive panel of known ONP and SGN-like cell markers during the time course of hDPSCs differentiation. Atomic force microscopy revealed that hDPSC-derived SGN-like cells exhibit similar nanomechanical properties as their in vivo SGN counterparts. Furthermore, spiral ganglion neurons from newborn rats come in close contact with hDPSC-derived ONPs 5 days after co-culturing. Our data demonstrate the capability of hDPSCs to generate SGN-like neurons with specific lineage marker expression, bipolar morphology, and the nanomechanical characteristics of SGNs, suggesting that the neurons could be used for next-generation cochlear implants and/or inner ear cell-based strategies for SNHL.

## 1. Introduction

Sensorineural hearing loss (SNHL) is the most common type of hearing loss. Auditory neuropathy spectrum disorder, defined by damage to the spiral ganglion neurons (SGNs) with relative preservation of the hair cells (HCs), is the cause of significant hearing impairment due to the lack of transmission of the signal from the sensory organ (cochlea) to the brain. While the deficit in HCs can be functionally overcome by a cochlear implant, no treatment is currently available for SGN loss. Without neurons, most of the currently available cochlear implants will not function [1]. A potential therapeutic approach to auditory neuropathy would be to replace the sensory neurons by transplantation of exogenous, in vitro-maintained, stem cell-derived otic neuronal progenitors (ONPs). The transplanted cells could also provide a means of delivering supportive neurotrophic factors to promote the survival of remaining neurons. Moreover, the delivery of ONPs at the time of cochlear implantation could extend the applicability and success rate of the current cochlear implant approach. The proof of concept has been established in a previous study [2], showing that human embryonic stem cells can produce ONPs, and that these cells can partially repair a damaged cochlear nerve in vivo. However, to develop a cell therapy to replace SGNs, the in vitro generation of appropriate ONPs and SGN-like cells from reliable and easily accessible sources of adult-tissue stem cells is required. Dental pulp-derived human mesenchymal stem cells (hDPSCs) could be an alternative source of cells, as they possess both mesenchymal and neural features due to their ectodermal origin [3,4], allowing them to self-renew and differentiate into multiple cell types (reviewed in [5]). The hDPSCs could have additional advantages when compared to adult stem cells from other sources, as dental pulp epithelium and cochleo-vestibular ganglions have relatively close embryonic development and share some signaling pathways with the neural crest [6]. Furthermore, tooth tissue is easily acquired from biological waste from avulsions of wisdom teeth or premolars [7]. These characteristics make hDPSCs potential donor cells for cell therapy and/or to provide replacement otic neurons to improve the performance of cochlear implants.

During inner ear development, the neurosensory cells are derived from the otic vesicle [8], which is induced from the non-neural ectoderm by a lateral-to-medial gradient of bone morphogenetic protein (BMP) [9]. The ventral region contains otic neuronal progenitors that give rise to SGNs (reviewed in [10,11]). Previous studies demonstrated that Sonic hedgehog (Shh) and retinoic acid (RA) synergistically promote the expression of sensory neuron markers and facilitate otic sensory neuronal differentiation [12]. It was also shown that Shh- and BMP-signaling and supplementation of neurotrophic factors (i.e., brain-derived neurotrophic factors BDNF and NT3) play an important role in the generation of SGN-like neurons from pluripotent stem cells [2,13,14,15]. Despite interest in the use of mesenchymal stem cells to generate SGN-like cells for the appropriate properties that they offer, in vitro differentiation of SGN-like neurons from adult hDPSCs through the early otic lineage has not yet been demonstrated.

In the current study, we assessed the possibility of deriving ONPs and SGN-like cells by stepwise-guided differentiation of hDPSCs. We first characterized the sphere-forming capacity and initial otic neuronal induction, followed by two differentiation paradigms, to screen factors, signals and substrate matrices that can promote otic neuronal lineage and SGN-like cells. We then characterized cells from each paradigm at the cellular and molecular levels and by the biomechanical evaluation of differentiated SGN-like cells. Finally, we explored the potential of neuronal connection of human ONPs with rat SGN explants in a co-culture system.

## 2. Results

### 2.1. Differentiation of hDPSC-Derived Early Otic/Placodal Progenitor Cells

**Figure 1:**
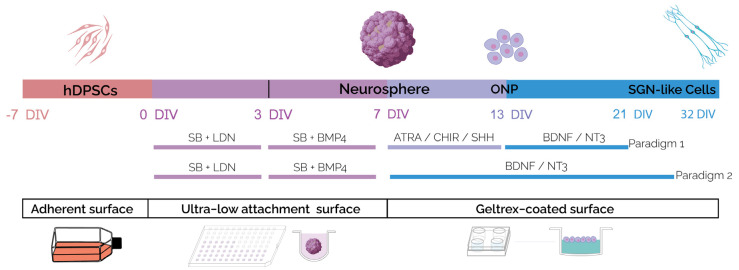
Schematic illustration outlining the timeline and conditions of the newly established protocol for the generation of hDPSC-derived SGN-like cells. The hDPSCs were exposed to SB/LDN for 4 days in vitro, followed by treatment with SB/BMP4 for an additional 3 days in vitro to generate neurospheres in ultra-low attachment (ULA) plates. The generated neurospheres were then plated on a Geltrex-coated surface and differentiated following differentiation paradigms 1 or 2. For paradigm 1, neurospheres were exposed to ATRA/SHH/CHIR until 13 DIV and then to BDNF/NT3 until 21 DIV. For paradigm 2, neurospheres were only exposed to BDNF/NT3 for the same period in vitro. The culture period was extended to 32 DIV in paradigm 2 to assess the effect of a prolonged in vitro maturation. Abbreviations: DIV: day in vitro; SB: SB431542: TGFb inhibitor; LDN: LDN-193189: bone morphogenetic protein (BMP) pathway inhibitor; BMP4: bone morphogenetic protein 4, ATRA: all trans retinoic acid; SHH: Sonic hedgehog; BDNF: brain-derived neurotrophic factor; NT3: neurotrophin 3.

We devised a new in vitro strategy to generate SGN-like cells by a stepwise differentiation of hDPSCs (Figure 1). Previous studies reported the requirement of dual BMP activation/inhibition to initiate early otic/placodal differentiation from human pluripotent stem cells [13,14,16,17]. Therefore, we first cultured floating hDPSCs in an LDN/SB- and BMP/SB-supplemented differentiation medium, to test the impact of the modulating pathways involved in otic development on hDPSC differentiation towards a human otic placodal-like cells phenotype under a neurosphere assay.

Before starting the differentiation procedure, we confirmed that the isolated hDPSCs displayed flattened and fibroblastic shape and were virtually all STRO-1 immuno-positive (Figure S1A) [18,19,20]. Moreover, these cells are positive for known mesenchymal antigens i.e., CD73, CD90, and CD105 (Figure S1B), and have a potential for adipogenic and osteogenic differentiation (Figure S1C–F). When cultured under floating conditions, hDPSCs aggregate into neurospheres (Figure S2A) and show a proliferative activity, characterized by an increase in the number of cells/neurosphere (Figure S2B), a population-doubling time (Figure S2C), and Edu staining (Figure S2D) during the time course of differentiation.

**Figure 2:**
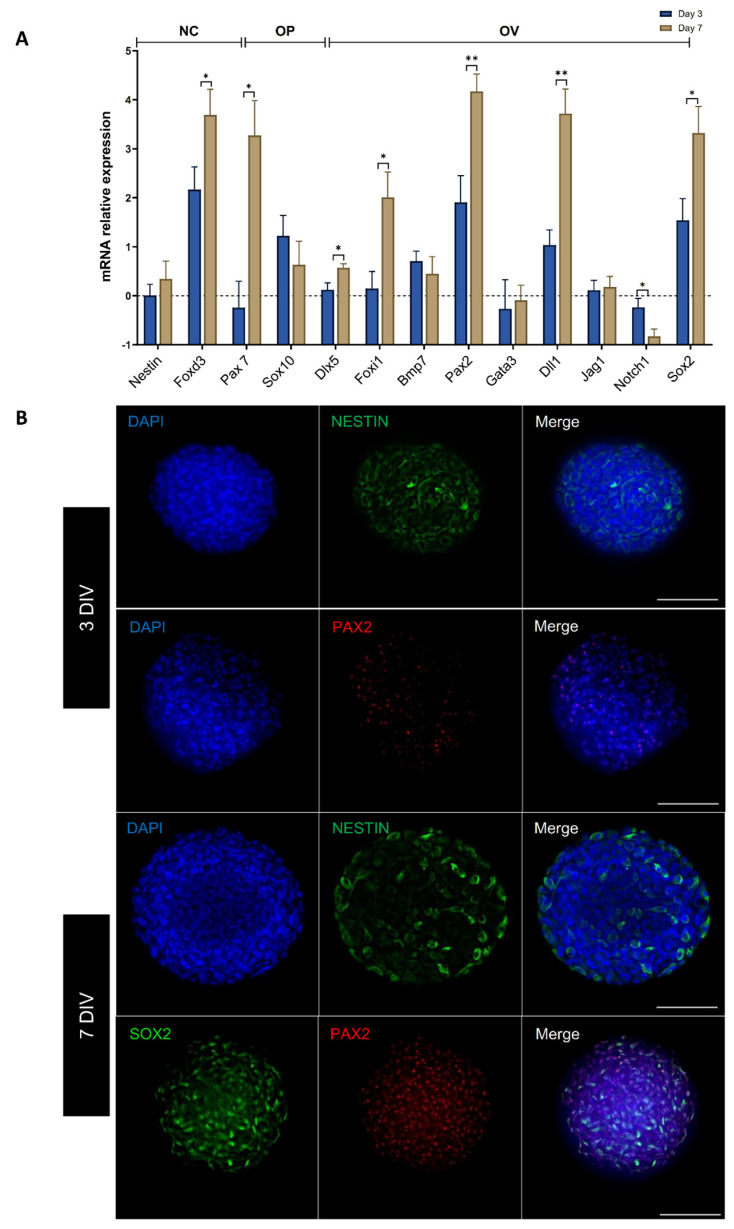
Induction of early neuronal and otic/placodal markers expression in hDPSC-derived neurospheres. (**A**) Bar chart showing relative gene expression levels in logarithmic (Ln) scale, obtained by qPCR analyses for three distinct panels of genes featuring the neural crest, otic placode, and otic vesicle, respectively. Cells were collected at 3 and 7 DIV of differentiation and analyzed to assess the effects of the dual inhibition/activation of BMP-signaling under continuous TGFb inhibition on otic induction. Noticeably, the results demonstrate a significant upregulation of otic/placode (*Dlx5*) and otic/vesicle markers (*Pax2*, *Sox2*). Bars represent SD. Statistically significant differences are indicated by * *p* ≤ 0.05, ** *p* ≤ 0.01 (one-way ANOVA), *n* = 3 independent experiments. Abbreviations: NC: neural crest; OP: otic placodal; OV: otic vesicle. The dashed line represents gene expression of undifferentiated hDPSCs. (**B**) Immunocytochemistry shows the expression of NESTIN and PAX2 during the time course of in vitro differentiation. A large proportion of cells within the neurospheres are NESTIN immunopositive at 3 and 7 DIV (shown in green), while PAX2 expression (shown in red) was principally observed at 7 DIV, and PAX2 immunopositive cells co-expressed SOX2. Cell nuclei were counterstained with DAPI (blue). Scale bars = 100 µm.

To specifically assess the fate of differentiated cells during the early phase of differentiation, cells were either collected or fixed at 3 and 7 DIV and analyzed in parallel by qPCR and immunocytochemistry for specific cell-type markers of neural crest (NC) and otic lineage, i.e., otic placode (OP), otic vesicle (OV) (Figure 2A,B), and neural progenitors (Figure S3). We explored the dynamic expression of a comprehensive panel of NC, OP, and OV gene markers. Noteworthy, the qPCR analyses showed that treatment with SB/LDN for 3 days and then SB/BMP4 for 4 days led to a robust expression of NC progenitors (*Pax7*, *FoxD3*) and OP/OV (*Pax2*, *Foxi1*, *Dll1*) markers (Figure 2A). During inner ear development, OP induction is followed by OV formation (reviewed in [8,10,11,21]). The OP marker expression analysis at 7 DIV of differentiation revealed a significant upregulation of genes, such as Dlx5, which are among key otic/placodal markers. Importantly, a significant upregulation of OV-associated genes (i.e., *Pax2*, *Sox2*) was detected at 7 DIV when compared to undifferentiated cells (Figure 2A). In addition, neural crest markers, such as *FoxD3* and *Pax7*, were significantly higher at 7 DIV when compared to the 3 DIV cultures.

To support the qPCR results, we also studied the expression of neural crest (NESTIN) and OV (SOX2 and PAX2) markers by immunohistochemistry. We observed that the NESTIN immunoreactivity within the neurospheres was similar between 3 and 7 DIV.

The results showed that about 95% of cells remained immunopositive for the expression of NESTIN in neurosphere-derived hDPSC sub-populations (Figure 2B). The expression of PAX2 was detected at 3 DIV, indicating its early expression, and both SOX2 and PAX2 were detected and colocalized at the nuclear level in a subset of cells by 7 DIV (Figure 2B and Figure S4). These examples of immunolocalization of NC and OP/OV markers at days 3 and 7 in differentiated neurospheres support the overall data from the qPCR analysis. Altogether, these data demonstrate that the differentiated cells derived from hDPSCs rapidly engaged toward OP/OV cell fate by 7 DIV upon SB/LDN and SB/BMP4 treatment using a 3D neurosphere culture.

### 2.2. Enrichment of DPSC-Derived Cells Expressing ONP and SGN Early Markers

Previous studies have demonstrated the roles of Shh, retinoic acid and WNT pathways in otic neuronal development in vivo [22,23,24,25]. Additionally, two crucial neurotrophins (i.e., BDNF and NT-3) and their associated receptors, TrkB and TrkC have been shown to be necessary for the normal development of afferent innervation during early human inner ear development [11,26,27]. All of these pathways and neurotrophins can also enhance the production of SGN-like cells from pluripotent stem cells in mammalian inner ear organoids [13,14,16,28]. We therefore tested the effects of exposing the 7 DIV neurospheres to small molecules that modulate these pathways under two in vitro differentiation paradigms. In paradigm 1, the neurospheres were exposed to ATRA/SHH/CHIR until 13 DIV and then to BDNF/NT3 until 21 DIV, while under paradigm 2, they were only exposed to BDNF/NT3 for the same period (Figure 1).

**Figure 3:**
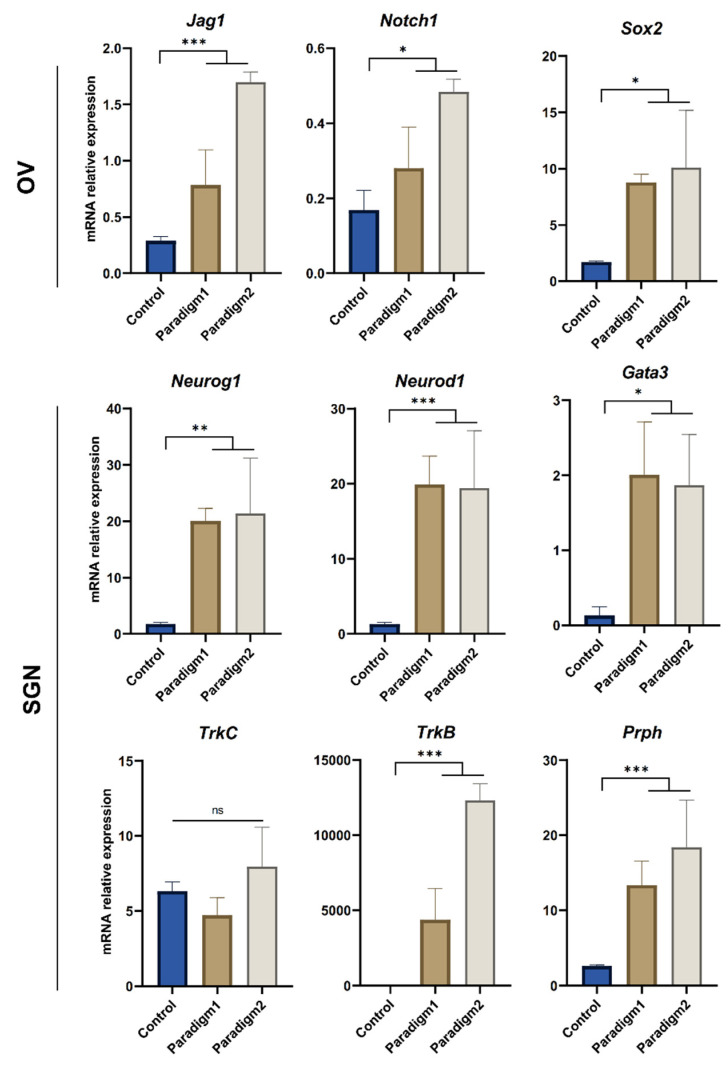
Analyses of otic vesicle and SGN gene markers in differentiated cells at 21 DIV. Bar charts showing relative gene expression levels were obtained by qPCR analyses of genes featuring OV and SGN lineages in cells differentiated under paradigms 1 and 2, as compared to control culture conditions. The differentiated cells were collected at 21 DIV from 3 independent culture experiments. A significant upregulation of OV and SGN markers was observed in both paradigms when compared to the control culture condition. Moreover, paradigm 2 showed the most significant upregulation of a subset (*Prph*, *TrkB*) related to both OV and SGN lineage. Statistical differences were determined with one way ANOVA. *p* values are indicated with * *p* ≤ 0.05, ** *p* ≤ 0.01, *** *p* ≤ 0.001, ns: not significant.

Investigating the variations in the relative expression levels of some specific lineage gene markers allowed for a comparison between the neuronal induction paradigms 1 and 2 at 13 DIV, revealing that the main difference was related to the relative expression levels of a subset of ONP markers (Figure S5). At 13 DIV, both paradigms induced the expression of the ONP markers, such as *Bmp7*, *Pax2*, *and Sox2*. However, paradigm 2 induced a significantly higher expression of *Neurod1* and *Neurog1*, suggesting a robust early commitment towards SGN-like phenotype under this culture paradigm. This suggestion could be supported by the significant downregulation of the expression of *Nestin* and *Dlx5*, which are related to early otic/placode progenitors. At 21 DIV, qPCR analysis of differentiated cells from both culture paradigms (Figure 3) showed strong evidence of the otic neuronal differentiation process. The relative expression of a panel of OV-related gene (i.e., *Sox2*, *Notch1*, *Jag1*) markers was significantly higher in both paradigms when compared to their expression levels under control conditions. The relative expression of the SGN-related gene markers (i.e., *Neurog1, Neurod1*, *Gata3*) was also significantly higher under the two culture paradigms, as compared to their expression levels under control conditions. Furthermore, we noticed that the overall relative expression levels were higher in paradigm 2 when compared to the paradigm 1 culture, as related to the relative expression of two key SGN (i.e., *TrkB*, *Prph*) markers. These results are in line with the previous analysis conducted at 13 DIV of differentiation and support the efficient commitment towards otic neuronal cell fate under the paradigm 2 differentiating-culture condition.

**Figure 4:**
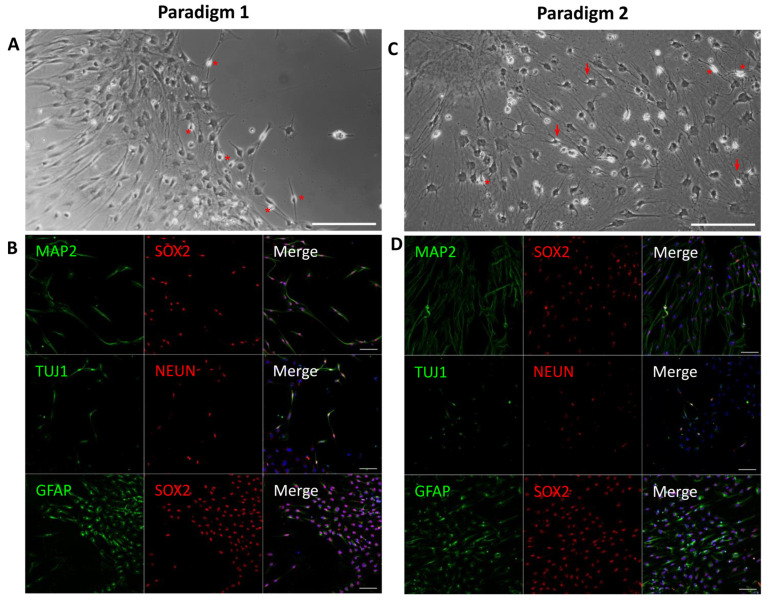
Representative images of immunocytochemical analyses of the expression of neural markers at 21 DIV in paradigm 1 and 2 cell cultures. (**A**) Phase-contrast image showing hDPSC-derived otic neuronal progenitors in paradigm 1 cultures. Differentiated cells have a bipolar morphology, with a round soma (asterisks), in a dense network. (**B**) The immunostaining shows the expression of MAP2/TUJ1/GFAP in the cytoplasm (shown in green) and SOX2/NEUN at nuclear level (shown in red) in paradigm 1 cultures. (**C**) Phase-contrast image showing hDPSC-derived otic neuronal progenitors in paradigm 2 cultures. Among differentiated cells, some have a bipolar morphology (asterisks), while others display a phenotype close to glial cells (arrows). (**D**) The immunostaining shows expression of MAP2/TUJ1/GFAP in the cytoplasm (shown in green) and SOX2/NEUN in the nuclei (shown in red) in paradigm 2 cultures. Under this paradigm, more double immunopositive SOX2/GFAP cells differentiated were detected, as compared to paradigm 1. DAPI staining is shown in blue. Scale bars = 100 μm in all panels.

**Figure 5:**
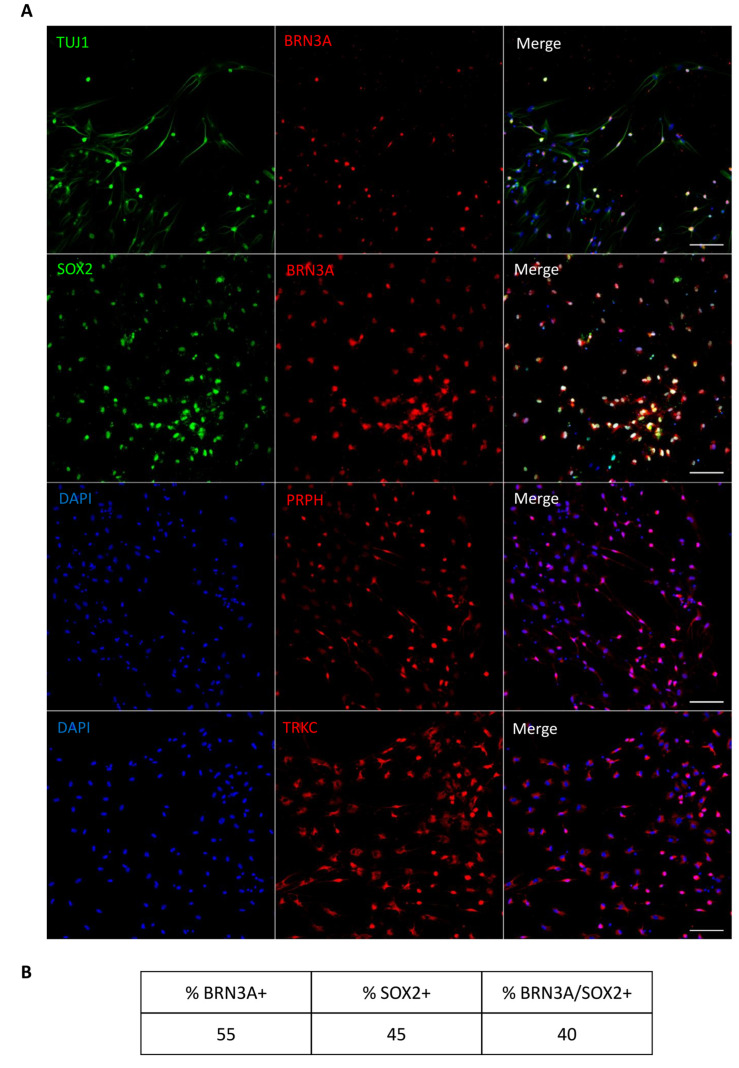
Characterization of the expression of SGN markers at 32 DIV in paradigm 2. (**A**) The differentiated SGN-like cells displayed a bipolar morphology and expressed a panel of SGN known markers such as SOX2 (green), BRN3A (red), TUJ1 (green), PRPH (red), and TRKC (red). A subset of these differentiated cells was double immunopositive for SOX2/BRN3A, characteristic of SGN phenotype. In addition, they express TRKC and PRPH which are also SGN-related markers. Scale bar = 100 µm. (**B**) The table represents the percentage of cells expressing SOX2, BRN3A, and SOX2/BNR3A. Cell count indicates about 40% of the differentiated cells at 32 DIV are double immunopositive for BRN3A/SOX2 from n = 2709 counted cells.

We next examined the expression of a subset of mature neuronal lineage (i.e., TUJ1, MAP2, NEUN, SOX2) and SGN-related (i.e., BRN3A) markers on differentiated cells from both paradigms at 21 DIV by immunohistochemistry (Figure 4) and quantified the percentage of immunopositive cells (Figure S6). In both paradigms, the differentiated cells expressed neuronal markers such as NEUN, TUJ1, and MAP2 (Figure 4B,D), but we could not detect the expression of BRN3A, which is a key transcription factor of the initial SGN differentiation process. When the differentiation period was extended to 32 DIV (Figure 5A and Figure S7), we observed that only the cultures differentiated under paradigm 2 were maintained, with good cell viability in comparison to the cells differentiated in paradigm 1. The prolongation of the cultures to 32 DIV of paradigm 2 led to the differentiation of a subset of SGN-like cells with a bipolar phenotype. Interestingly, about 40% of these bipolar SGN-like cells were double immunopositive for BRN3A/SOX2 (Figure 5B), in addition to expressing other otic neuronal markers (i.e., TUJ1, PRPH, and TRKC).

### 2.3. AFM Characterization Reveals Similarities between SGNs In Vitro and SGNs In Vivo

**Figure 6:**
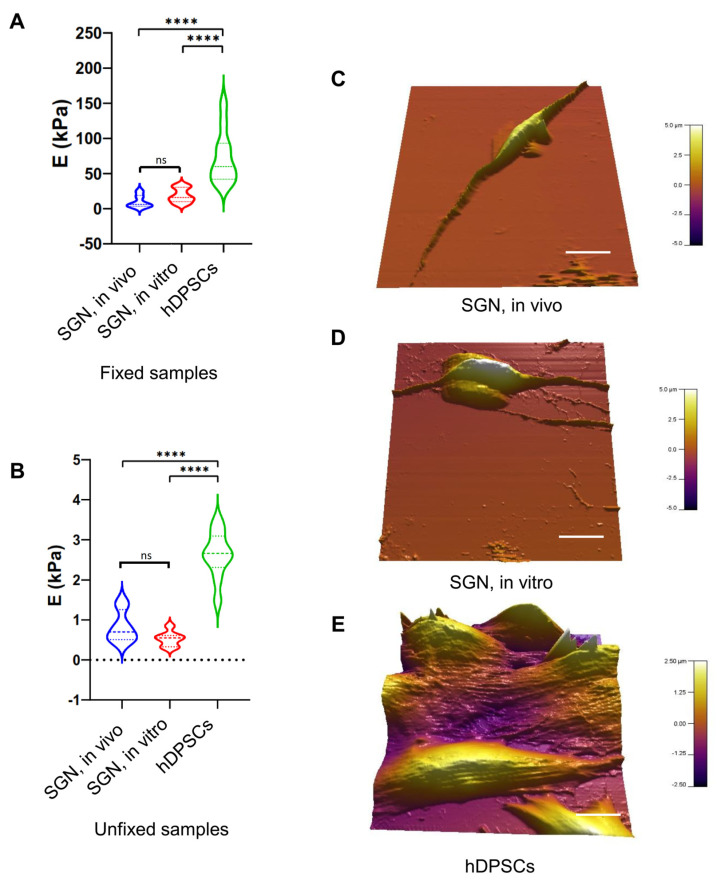
AFM-based nanomechanical characterization of SGN-like cells at 32 DIV. (**A**) Violin plot of measured Young’s modulus (**E**) of fixed SGN in vivo, SGN in vitro, and undifferentiated hDPSCs. A significant difference was observed between hDPSCs and SGN samples. However, both SGN in vivo and in vitro were similar related to Young’s modulus measurements. (**B**) Violin plot of measured Young’s modulus of unfixed SGN in vitro, SGN in vivo, and undifferentiated hDPSCs. A significant difference was also observed between hDPSCs and SGN samples. Young’s modulus measurements of SGN in vivo and in vitro were similar. These measurements indicate strong similarities between SGN in vivo and SGN in vitro differentiated from hDPSCs at the nanomechanical level. (**C**) Three-dimensional reconstruction of analyzed SGNs in vivo, showing a bipolar morphology. (**D**) Three-dimensional reconstruction of analyzed SGNs in vitro, showing their bipolar morphology acquired after the in vitro differentiation process, which is different form the morphology of hDPSCs shown in (**E**). (**E**) Three-dimensional reconstruction of analyzed hDPSCs showing the characteristic elongated morphology of these cells. Statistical differences were determined with one-way ANOVA. *p* values are indicated with **** *p* ≤ 0.0001. n = 10 measurements. Scale bar = 20 µm.

To add a new dimension of characterization, we explored the nanomechanical properties of our generated SGN-like cells derived from the culture paradigm 2 at 32 DIV and compared them to in vivo SGNs isolated from rat pups and to undifferentiated hDPSCs. The results showed that both the SGN-like cells and in vivo SGNs share the same Young’s modulus in either fixed samples (5–10 KPa) (Figure 6A) or unfixed samples (~0.5–0.7 KPa) (Figure 6B). These measurements of Young’s modulus were significantly lower than the measurements from hDPSCs, which were much stiffer.

Interestingly, the topographic reconstructions revealed that both in vivo SGNs and in vitro SGNs share a bipolar elongated morphology (Figure 6C,D), whereas hDPSCs are characterized by their characteristic elongated fibroblastic morphology (Figure 6E). The contrast in morphology between in vitro SGN-like cells and hDPSCs, in addition to the significant difference between their nanomechanical properties, constitute another evidence for the SGN-like cells generation from the stepwise differentiation of hDPSCs.

### 2.4. Spiral Ganglion Neurons from Newborn Rats Send Out Neurites That Contact ONPs

**Figure 7:**
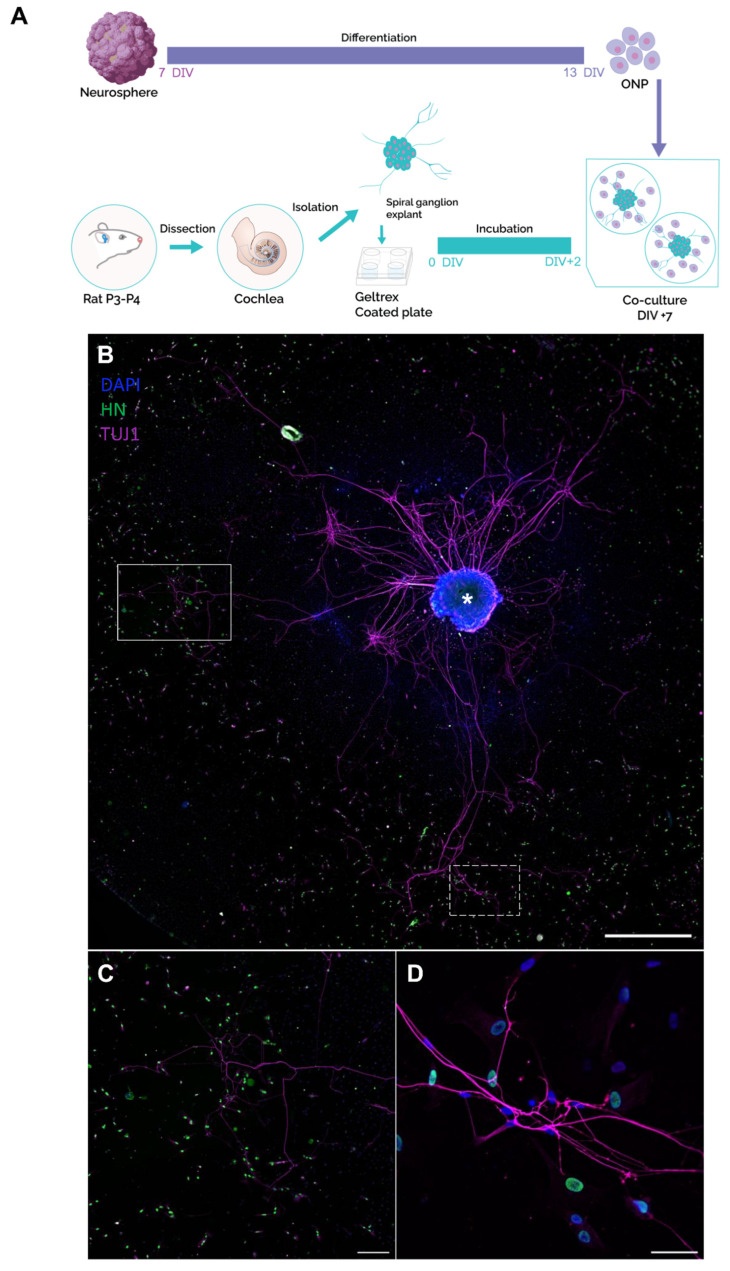
Characterization of the co-cultures between human ONP cells and rat SG explants. (**A**) Schematic representation of different steps of the co-culture procedure. Neurospheres were generated from hDPSCs and differentiated to ONP cells until 13 DIV. In parallel, cochlear explants were dissected from the inner ear of postnatal day P3 rats, followed by SG isolation and culture on Geltrex-coated plates for 48 h. Then, the ONP cells were detached from the substrate and co-cultured with SG explants for 5 additional days. (**B**) Representative image of neurite outgrowths from SG explant (asterisk) immunostained with anti-TUJ1 (shown in magenta), projected towards the ONP cells, immunostained with anti-human nuclei (shown in green). DAPI was used to counterstain the nuclei. Scale bar = 1000 µm. (**C**) Magnification of the area indicated by the white rectangle in (B, showing outgrowth neurites (magenta) emanating from the SG explant towards ONP cells (green), Scale bar = 500 µm. (**D**) Magnification of the area indicated by the dashed rectangle in (**B**) highlights contacts between neurites from SG explant and the membrane of ONP cells. Scale bar = 50 µm. Abbreviations: ONP: otic neuronal progenitors; SG: spiral ganglion.

**Figure 8:**
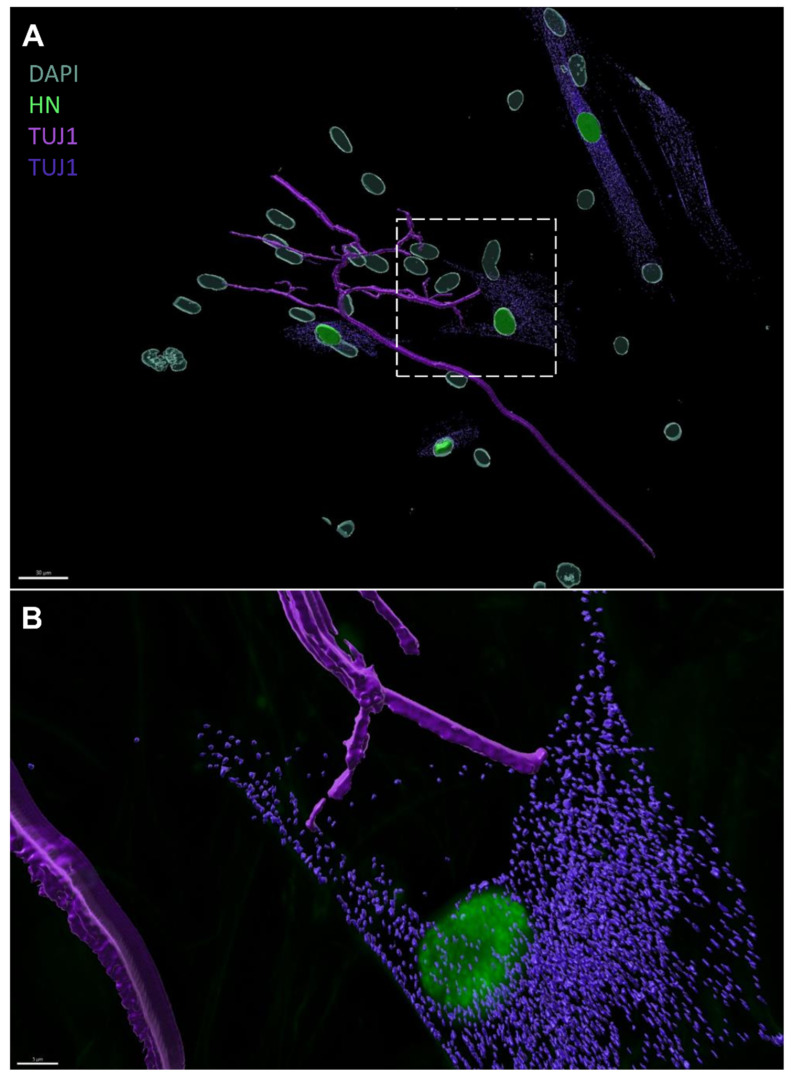
Three-dimensional reconstruction of SG explant-emanating neurites and ONP contacts using IMARIS. Figure S8B was used for the reconstruction with two look-up tables for TUJ1 staining. (**A**) Image represents the 3D reconstruction of neurite contacts between SGNs from the SG explant and ONP cells using IMARIS 10 software. The neurites (magenta = TUJ1 immunostaining) establish direct contacts with the membrane of ONP derived from hDPSCs (green: human nuclei, and purple: TUJ1). Scale bar = 30 µm. (**B**) Magnification of the area indicated by the square in (A) representing the image reconstruction by IMARIS of the neurites which established contacts during the co-culture. Scale bar = 10 µm.

To investigate the application potential of our in vitro-generated ONPs in restoring SGNs, we co-cultured dissociated ONPs from the paradigms 1 and 2 at 13 DIV, with the spiral ganglion (SG) explants isolated from rat pups (Figure 7A). We observed the neuronal outgrowths of SGNs (Figure 7B, Figures S8A and S9A), which were directed towards the ONPs (Figure 7C,D, Figures S8B and S9A). In some co-culture experiments, this neurite extension was beyond 1000 µm, extending from the core of the SGs to distant ONPs. Moreover, we noticed that these neurites establish contacts with the ONPs at the level of their membranes. To confirm these newly established contacts between ONPs and the neurites projecting from the SG explants, we performed a 3D reconstruction using Imaris 10 software. Interestingly, we were able to clearly observe the neurites that were in direct contact with the membranes of the ONPs (Figure 8A,B), which suggest the potential of ONPs to reconnect with the SG extracted from the inner ear of the postnatal rats.

## 3. Discussion

SNHL can be caused by primary degeneration of SGNs or secondary degeneration of these neurons after HC loss [29,30,31]. The replacement and maintenance of SGNs would therefore be an important step in any attempt to restore the auditory function in patients with damaged sensory neurons or HCs (reviewed in [32,33]). The successful replacement of lost or damaged SGNs will likely result in improved clinical outcomes for cochlear implant recipients. Human dental pulp represents an easily accessible source to isolate stem cells from, with low invasiveness and in substantial quantities [3,34], and could therefore facilitate the development of a clinically viable, cell-based cell therapy for SNHL. Considering the future potential applications of hDPSCs, the focus of the present study was to develop optimal culture conditions for their differentiation into ONPs and SGN-like cells via neurosphere-mediated and direct otic neuronal induction on a hydrogel matrix. During the time course of these two steps of in vitro differentiation procedures, we tracked the emergence of OP/OV, ONPs, and SGN-like phenotypes.

We reasoned that, similar to the developmental in vivo situation, a timely directed modulation of key signaling pathways could promote ONP and initial SGN lineage from hDPSCs-derived neurospheres. To test this hypothesis, we challenged neurospheres by modulating the Wnt/Shh/RA pathways under NT3/BDNF exposure (paradigm 1) or with NT3/BDNF only (paradigm 2). We found that both in vitro paradigms yielded the differentiation of hDPSC-derived ONPs and SGN-like cells at 13 and 21 DIV, respectively. However, the most significant upregulation of OV and SGN lineage markers was observed under paradigm 2 differentiating condition. This finding suggests that a strong commitment toward otic neuronal lineage as Neurod1 is a major transcription factor expressed with TrkB during SGN early specification [35,36]. It is also well established that BDNF and NT3, with their respective TrkB and TrkC receptors, are implicated in cochlear innervation during inner ear morphogenesis [37].

In addition to the difference in the expression pattern of OV and SGN markers, another distinction between the culture paradigms 1 and 2 is related to their ability to progress with in vitro differentiation beyond 21 DIV. Only the cells differentiated under paradigm 2 continued to grow and retain a substantial survival by 32 DIV. These observations may be related to the high number of GFAP immunopositive cells in the culture paradigm 2 which represents about 40% of the cells (Figure S6). This subpopulation of GFAP immunopositive cells may include Schwann cell precursors or glial cells, and would represent a cell population also derived from hDPSC differentiation [38,39]. It is well known that glial and Schwann cells play an important role in the neurotrophic support and the survival of SGNs during inner ear development [40,41], which can explain the extended maturation of the cell cultures in paradigm 2 as compared to the cultures in paradigm 1. The SGN-like cells at 32 DIV displayed a bipolar phenotype close to mammalian auditory neurons grown in vitro. This neuronal bipolar morphology could be enhanced by the use of Geltrex substrate coating, as reported in the case of neuronal differentiation from pluripotent stem cells [42]. SGN-like cells are characterized by the co-expression of both SOX2 and BRN3A. This expression of these two otic neuronal transcription factors, which was observed only in the cells differentiated in paradigm 2, may indicate a possible early differentiation of some otic glutamatergic neurons [43]. In addition to BRN3A, 32 DIV SGN-like cells expressed PRPH and TUJ1, which may indicate the emergence of Type1 SGN precursors, supported by the previous expression of *NeuroD1* [44,45].

Additionally, we explored the nanomechanical properties of the generated SGN-like cells and compared them to in vivo SGN equivalents and to undifferentiated hDPSCs. Such nanomechanical characterization offers a high resolution cartography and meaningful information about the stiffness of biological samples (reviewed in [46]), allowing to distinguish between different cell populations, including the neuronal cell types. Our AFM analysis revealed that in vitro SGN-like cells and in vivo SGNs share the same Young’s modulus, and thus have the same nanomechanical properties, which are different from hDPSCs. Our initial characterization is consistent with a recent report that explored the optimal matrix mechanical properties to generate otic neurosensory progenitors, which is around 3 KPa [47], suggesting that our in vitro SGN-like cells are similar to their in vivo SGN counterparts. These findings also correlate with previous results [13], reporting that Geltrex coating is an adequate matrix to support SGN culture and the in vitro differentiation of these cells from stem cells. Altogether, these data support the efficiency of our protocol to generate SGN-like cells from hDPSCs under the differentiating culture condition of paradigm 2. They also provide new insights for the use of nanomechanical characterization to assess the state of in vitro stem cell differentiation. By sharing the same cytoskeletal properties (reviewed in [48]) with in vivo SGNs, we expect an enhanced integration of SGN-like cells in autologous engraftment for auditory nerve recovery.

To assess the therapeutic potential of in vitro-generated human ONPs, we investigated their neurite growth capacity to form neuronal connections with rat postnatal SGNs under a co-culture assay. Such co-culture experiments revealed a preferential projection of SGNs towards the ONPs, establishing direct contacts. This suggests that ONPs might provide attractive guidance cues to SGNs, which could offer therapeutic benefits in the context of preservation or regeneration of neuronal contacts from SGNs. These attractive signals could probably be related to the fact that ONPs may retain a secretome feature memory of the hDPSCs from which they derive. For instance, the secretome of hDPSCs is reported to be very rich in neurotrophic and growth factors that promote neuronal differentiation [49,50]. Furthermore, a recent report showed a similar effect of neuronal projections of SGNs due to secreted factors from otic pericytes in a co-culture system [51]. This effect correlates to some extent with our observations, given that the pericyte subpopulation resides in the dental pulp tissue and contributes to the homeostasis of teeth (i.e., nervous and vascular systems) [52,53]. Future studies are required to explore the nature of these contacts, the presence of synaptic markers, the electrophysiological properties of these SGN-like cells, and whether trophic factors are secreted by ONPs.

## 4. Materials and Methods

### 4.1. Collection of Human Dental Pulp and Culture

The human dental pup stem cells (hDPSCs) were isolated from extracted wisdom teeth from three young healthy patients (14, 18, and 21 years old) (Appendix A). Informed consent was obtained from the patients after receiving approval by the local ethics committee (Comité de protection des Personnes, Centre Hospitalier de Montpellier). We used a previously established protocol to recover pulp cells [7]. From each patient, one to two wisdom teeth were used. Briefly, the teeth were cleaned with 2% chlorhexidine, then cut at the cementum–enamel junction by using a sterilized drill. The teeth were broken into two pieces with a scalpel and the pulp was recovered from their cavities by using tweezers. Pulps were first cut into small pieces then digested in a 2 mL solution of 3 mg/mL collagenase type 1 and 4 mg/mL of dispase (Corning, Corning, New York, NY, USA) for 1 h at 37 °C. The cell suspension was filtered using a 70 µm strainer (Falcon,, Corning, New York, NY, USA) and transferred to a T75 flask (Falcon, Corning, New York, NY, USA) containing 10 mL of complete medium: A-MEM (Gibco, Thermo Fisher Scientific, Waltham, MA, USA), 10% FBS (Sigma, Saint Louis, MO, USA), 100 µg/mL streptomycin (S) (Sigma, Saint Louis, MO, USA), and 100 U/mL penicillin (P) (Sigma, Saint Louis, MO, USA). Medium was first changed after 24 h, then every 3 days for 1 week. At confluency, the cells were passaged by washing the culture with DPBS (HyClone, Cytiva, Washington, DC, USA) and then detaching them using Trypsin-EDTA (Gibco, Thermo Fisher Scientific, Waltham, MA, USA).

### 4.2. Flow Cytometry

The immunophenotypic detection of mesenchymal stem cell markers, i.e., CD90, CD73, CD105, was performed by flow cytometry. The dental pulp cells at passage 4 were rinsed with DPBS (HyClone, Cytiva, Washington, DC, USA) and then detached by using Accutase (Sigma, Saint Louis, MO, USA). The cells were washed three times. Unspecific binding sites were blocked with a FACS solution consisting of 2% FBS (Sigma, Saint Louis, MO, USA) diluted in PBS for 45 min, at room temperature. The cells were then stained with anti-CD73-FITC (Invitrogen, Thermo Fisher Scientific, Waltham, MA, USA), anti-CD105-APC (Invitrogen, Thermo Fisher Scientific, Waltham, MA, USA) and anti-CD90-PE (Invitrogen, Thermo Fisher Scientific, Waltham, MA, USA) for 1 h, at 4 °C. The cells were washed 3 times with DPBS and kept in a FACS solution. Flow cytometry data acquisition was performed in Novocyte2 cytometer (Agilent, Agilent, Santa Clara, CA, USA) and data were analyzed with NovoExpress software (Version 1.6.0, Agilent, Santa Clara, CA, USA).

### 4.3. Multilineage Differentiation of hDPSCs

Dental pulp cells were seeded at a density of 105 cells/cm^2^ and cultured in a complete medium until confluency. For osteogenic cultures, the medium was composed of A-MEM (Gibco, Thermo Fisher Scientific, Waltham, MA, USA), supplemented with 15% FBS (Sigma, Saint Louis, MO, USA), 10^−8^ M dexamethasone (Sigma, Saint Louis, MO, USA), 50 µg/mL L-Ascorbate Phosphate (Sigma, Saint Louis, MO, USA), 5 mM B-Glycerphosphate (Sigma, Saint Louis, MO, USA), and 1.8 mM Monopotassium Phosphate (Sigma, Saint Louis, MO, USA). The medium was changed twice a week during 3 weeks [54]. For adipogenic cultures, the complete medium was replaced by A-MEM (Gibco, Thermo Fisher Scientific, Waltham, MA, USA) supplemented with 10% FBS (Sigma, Saint Louis, MO, USA), 10^−5^ M dexamethasone (Sigma, Saint Louis, MO, USA), 50 µg/mL L-Ascorbate Phosphate (Sigma), 1 µg/mL Insulin (Sigma, Saint Louis, MO, USA), and 0.5 mM isobutylethylxantine (Sigma, Saint Louis, MO, USA) [55]. Control age-matched cultures were maintained with a complete medium in parallel with the differentiated cultures for a total of three weeks. The mineralization for osteogenic differentiation was assessed by Alizarin Red staining (Sigma, Saint Louis, MO, USA), whereas adipogenic differentiation was assessed by Oil Red O staining (Sigma, Saint Louis, MO, USA).

### 4.4. Three-Dimensional Floating Sphere Culture Assay

The hDPSCs at passage 4 were detached using Trypsin-EDTA (Gibco, Thermo Fisher Scientific, Waltham, MA, USA). About 20,000 cells per well were cultured in a ultra-low attachment 96-well plate (Nunclon Sphera, Thermo Fisher Scientific, Waltham, MA, USA) in a neurosphere culture medium (DFNBEb): DMEM/F12 (Gibco, Thermo Fisher Scientific, Waltham, MA, USA), 1× P/S staining (Sigma, Saint Louis, MO, USA), 1% N-2 supplement (Gibco, Thermo Fisher Scientific, Waltham, MA, USA), 1% B-27 supplement (Gibco, Thermo Fisher Scientific, Waltham, MA, USA), 20 ng/mL EGF (Gibco, Thermo Fisher Scientific, Waltham, MA, USA), and 20 ng/mL bFGF (Invitrogen, Thermo Fisher Scientific, Waltham, MA, USA), for 7 days. From day 1 up to day 3, the DFNBEb medium was supplemented with 1 µM SB431542 (Sigma, Saint Louis, MO, USA) and 1 µM LDN-193189 (Reprocell, Stemgent, Beltsville, MD, USA). From day 4 up to day 7 in vitro, the DFNBEb medium was supplemented with 1 µM SB431542 (Sigma, Saint Louis, MO, USA) and 10 ng/mL BMP4 (Reprocell, Stemgent, Beltsville, MD, USA). Half of the medium was replaced every 2 days, and the concentrations were adjusted to the final medium volume in culture wells.

### 4.5. Cell Proliferation

Neurosphere formation was monitored daily by taking pictures of selected wells with an inverted microscope (Axiovert A1, Carl *Zeiss*, Oberkochen, Germany)). Diameter measurements were performed by using the spheroid sizer tool in MATLAB (Version R2016a, Mathworks, Natick, MA, USA) [56]. Data analysis was performed with GraphPad Prism 8 (GraphPad Software, Boston, MA, USA). Proliferation within neurospheres was assessed by using Click-iT Plus EdU Alexa Fluor 594 kit (Invitrogen, Thermo Fisher Scientific, Waltham, MA, USA) to label the entire population of proliferating cells. Briefly, EdU was first added to the medium at day 0 and renewed at every medium replacement. In some experiments, EdU was added from day 3, and neurospheres were collected for immunochemistry analysis following the manufacturer’s protocol.

Population doubling time (*PDT*) was calculated at days 3 and 7 in vitro. For each experiment, 12 neurospheres were collected in a tube and dissociated with an Accutase solution (Sigma) to obtain a cell suspension. The cells were counted manually using KOVACS counting cells. The total number of cells was normalized to the number of neurospheres. The PDT was then calculated using the following formula [4]:

$${PDT}_{D0-D3}=\frac{{log}_{10}\left(2\right)\times ∆t}{{log}_{10}\left(N_{D3}\right)-{log}_{10}\left(N_{D0}\right)}$$

$${PDT}_{D0-D7}=\frac{{log}_{10}\left(2\right)\times ∆t}{{log}_{10}\left(N_{D7}\right)-{log}_{10}\left(N_{D0}\right)}$$

### 4.6. Otic Neuronal Induction

Otic neuronal induction was performed on Gletrex-coated surfaces. A 10% Gletrex (Gibco, ThermoFisher, Waltham, MA, USA) solution was prepared by diluting the hydrogel matrix in a culture medium. Sterile round glass coverslips placed in 4-well culture plates were covered with 180 µL of coating solution. The plates were placed in the incubator at 37 °C for 15 to 25 min for Geltrex polymerization. To induce neuronal differentiation, neurospheres at day 7 in vitro were plated on the pre-coated surface. We tested two neuronal induction paradigms for a total duration of 14 days in vitro. In paradigm 1, day 7 neurospheres were cultured in a DFNBEb medium: DMEM/F12 (Gibco, ThermoFisher, Waltham, MA, USA), 1× P/S (Sigma, Saint Louis, MO, USA), 1% N-2 supplement (Gibco, ThermoFisher, Waltham, MA, USA), 1% B-27 supplement (Gibco, ThermoFisher, Waltham, MA, USA), 20 ng/mL EGF (Gibco, ThermoFisher, Waltham, MA, USA), and 20 ng/mL bFGF (Invitrogen, ThermoFisher, Waltham, MA, USA) supplemented with 0.5 µM ATRA (Sigma, Saint Louis, MO, USA), 3 µM CHIR99021 (Reprocell, Stemgent, Beltsville, MD, USA), and 100 ng/mL SHH (R&D Systems Inc, Minneapolis, MN, USA) for 5 days, then cultured in a medium supplemented with 20 ng/mL BDNF (Peprotech Inc., Rocky Hill, NJ, USA) and 30 ng/mL NT3 (Peprotech Inc., Rocky Hill, NJ, USA) for an additional 8 days, up to day 21 in vitro. In paradigm 2, day 7 neurospheres were cultured for 14 days in a DFNB medium: DMEM/F12 (Gibco, ThermoFisher, Waltham, MA, USA), 1× P/S (Sigma, Saint Louis, MO, USA), 1% N-2 supplement (Gibco, ThermoFisher, Waltham, MA, USA), 1% B-27 supplement (Gibco, ThermoFisher, Waltham, MA, USA) supplemented with 20 ng/mL BDNF and 30 ng/mL NT3. In both paradigms, the medium was changed every 2 days. In some experiments, the differentiation period was extended up to 32 days. A control condition consisted of differentiating the neurospheres in a DFNB medium only for the same culture period.

### 4.7. Co-Culture Experiments of ONP Cells and SG Explants

Cochlear tissue for co-culture was isolated from postnatal day-3 rat pups. Six rat pups (12 cochleae) were used for each co-culture experiment. Following anesthesia on ice, the rat pups were decapitated, and the heads rinsed in 70% ethanol. Under sterile conditions, the skull was opened longitudinally, the temporal bone identified, and the bulla removed and placed into a chilled solution of explant media comprising 50 mL DMEM (Gibco, ThermoFisher, Waltham, MA, USA) and 10 mM HEPES (HyClone, Cytiva, Washington, DC, USA). We separated the spiral ganglion (SG) from the organ of Corti, and the isolated SG samples were maintained in chilled explant media until co-culture with ONPs. Co-cultures were set up by placing SG explants on Geltrex-coated wells for 48 h to ensure adhesion of the SG explants. The ONPs were then detached using Accutase and co-cultured with SG explants. Approximately 15,000 cells were used with each explant. Cultures were kept for 5 days in DMEM-F12 (Gibco, ThermoFisher, Waltham, MA, USA) medium with 10 ng/mL BDNF (Peprotech Inc., Rocky Hill, NJ, USA) and 10 ng/mL NT3 (Peprotech Inc., Rocky Hill, NJ, USA) [57]. Co-culture samples were maintained in the incubator at 37 °C, 5% CO_2_, and observed daily with a Zeiss inverted light microscope (Axiovert A1, Carl *Zeiss*, Oberkochen, Germany). After 5 days of co-culture, the samples were fixed and prepared for immunocytochemistry.

### 4.8. RNA Processing and qRT-PCR

Total RNA samples were collected from all stages investigated in vitro. For each stage, samples were collected from 3 biological triplicates. The cell samples were lysed with Trizol (Life Technologies, ThermoFisher, Waltham, MA, USA) and RNA purification was performed using a Zymo kit (R1050, Zymo Research, Irvine, Canada). RNA quantification was assessed with a Nano Drop 8000 spectrophotometer (Thermo Scientific, ThermoFisher, Waltham, MA, USA). cDNA synthesis was performed using ReadyScript™ cDNA Synthesis Mix (Sigma, Saint Louis, MO, USA) in a 20 µL final reaction volume, following the manufacturer’s protocol, and a Bio-Rad C1000 thermocycler according to the following program: 5 min at 25 °C, 30 min at 42 °C, and 5 min at 85 °C. For each sample, 400 ng of RNA was used as an RNA matrix for the reverse transcriptase enzyme. All qPCR reactions were carried out using 384-well plates in 3 technical replicates, with a 10 µL final reaction volume, on the LightCycler 480 System II (Roche, Basel, Switzerland). Reaction mix consisted of Power Track SYBR Green Master Mix 2X (Applied Biosystems, ThermoFisher, Waltham, MA, USA), specific primer pairs (final concentration 0.4–0.8 µM), 1 µL of 1:10 diluted cDNA per reaction, and H_2_O to a volume of 10 µL. The primer pairs used for gene expression analysis are listed in Supplementary Table S1. The PCR program consisted of an enzyme activation step at 95 °C for 2 min, followed by 45 cycles of qPCR reaction at 95 °C for 5 s and 60/64 °C for 30 s and finally a melting curve from 60 to 97 °C with 5 fluorescence acquisitions per °C. Expression levels were calculated by the comparative ΔΔCt method (2^−ΔΔCt^ formula), normalizing to the Ct-value of the RPS18 housekeeping gene. For the ΔΔCt calculation, expressions at day 0, representing undifferentiated hDPSCs, were taken as a reference. All values are presented as the mean ± standard deviation. Statistical significance for relative fold change values was determined using a one-way ANOVA (* *p* ≤ 0.05, ** *p* ≤ 0.01, *** *p* ≤ 0.001, **** *p* ≤ 0.0001).

### 4.9. Immunocytochemistry and Imaging

Culture samples at different stages of differentiation were fixed with 4% paraformaldehyde for 20 min then washed once with PBS and twice with Tris (Alfaeser, Haverhill, MA, USA) + 0.1% Triton (Sigma). Blocking and permeabilization steps were performed with 1% BSA and 0.5% cold fish gelatin (Sigma, Saint Louis, MO, USA) dissolved in a Tris + 0.1% Triton solution. Primary antibodies, listed in Supplementary Table S2, were diluted in blocking solution and incubated with the samples overnight, at room temperature. Then they were washed three times, followed by incubation with secondary antibodies for 45 min at room temperature. DAPI (Sigma, Saint Louis, MO, USA) was used to counterstain the cell nuclei. Image acquisition was performed on a Leica Thunder microscope. Images were processed with LasX software (Version 3.7.4.23463, Leica, Wetzlar, Germany). Cells were counted manually using ImageJ. Mosaics of imaged wells were used for cell counting. The fraction of immunopositive cells was reported to the total number of cells stained with DAPI (Sigma, Saint Louis, MO, USA in %. Graphs were established using GraphPad Prism8. For co-culture samples, IMARIS 10 software (v10.0, Bitplane, Victoria, Australia) was used for 3D reconstructions.

### 4.10. Nanomechanical Characterization by Atomic Force Microscopy (AFM)

The AFM force spectroscopy experiments were performed using an Asylum MFP-3D head coupled to a Molecular Force Probe 3D Controller (Asylum Research, Barbara, CA, USA). Triangular silicon nitride cantilevers (MLCT-NanoAndMore GmbH, Wetzlar, Germany), with a nominal spring constant of 10 pN/nm, were used. The spring constant of the cantilevers was determined using the thermal noise method available within the MFP-3D software (Version 16, Asylum Research, Barbara, CA, USA. Force-volume maps were performed in a PBS buffer (HyClone, Cytiva, Washington, DC, USA), at room temperature and with a maximum loading force of 1 nN, recording at least 10 maps per cell type. The recorded AFM-FS data were used to determine the Young’s modulus (E) of the tissue using a modified Hertz contact model Histograms of the distribution of Young’s moduli were fitted with a Gaussian function to obtain the mean E value of the probed cells. One-way ANOVA was used for comparison of Young’s modulus values. Tukey’s multiple comparisons were employed after performing normality tests for comparison of every mean with every other mean. Three cell types, including hDPSCs, native SGN (SGN, in vivo), and hDPSC-derived SGN-like cells (SGN, in vitro), were studied under live and fixed conditions. Statistical significance was set at *p* ≤ 0.05.

## 5. Conclusions

The hDPSCs are attractive candidates for regenerative medicine, as they offer an easily harvestable source for patient-specific therapeutics. In this study, we generated ONP- and SGN-like cells from hDPSCs following a reliable stepwise in vitro guidance procedure. The efficiency of this in vitro model was determined using a combination of approaches, including the expression of specific gene markers, analysis of cell morphology, and nanomechanical properties with AFM. Furthermore, using co-cultures, we demonstrated the potential of neuronal reconnection of hDPSC-derived ONPs with rat SGN explants. To our knowledge, this is the first report on the potential of hDPSCs to acquire in vitro features of auditory neurons, laying the foundation for additional studies to explore their engraftment potential in animal models of auditory neuropathy. Such studies will be essential in terms of clinical use and may be combined with cochlear implants to improve hearing.

## Data Availability

The original contributions presented in the study are included in the article and Supplementary Materials, further inquiries can be directed to the corresponding author.

## Supplementary Materials

The following supporting information can be downloaded at: https://www.mdpi.com/article/10.3390/ijms25169115/s1.

## Appendix A

For this work, dental pulp tissue, used to extract human dental pulp stem cells, was recovered from one to two wisdom teeth from each of the three donors. In total, more than 30 differentiation experiments were conducted and at least eight experiments were conducted with each batch of cells from a single donor. Data from at least three independent experiments were used for each section and qPCR data were pooled from all the donors.
