# Supplementary material for: Differentiation of Spiral Ganglion Neurons from Human Dental Pulp Stem Cells: A Further Step towards Autologous Auditory Nerve Recovery
Source: Int J Mol Sci. 2024 Aug 22;25(16):9115. doi: 10.3390/ijms25169115 (PMC11354632; doi:10.3390/ijms25169115)
Supplement: Supplementary file 1 [file ijms-25-09115-s001.zip › ijms-3083125-supplementary.pdf]

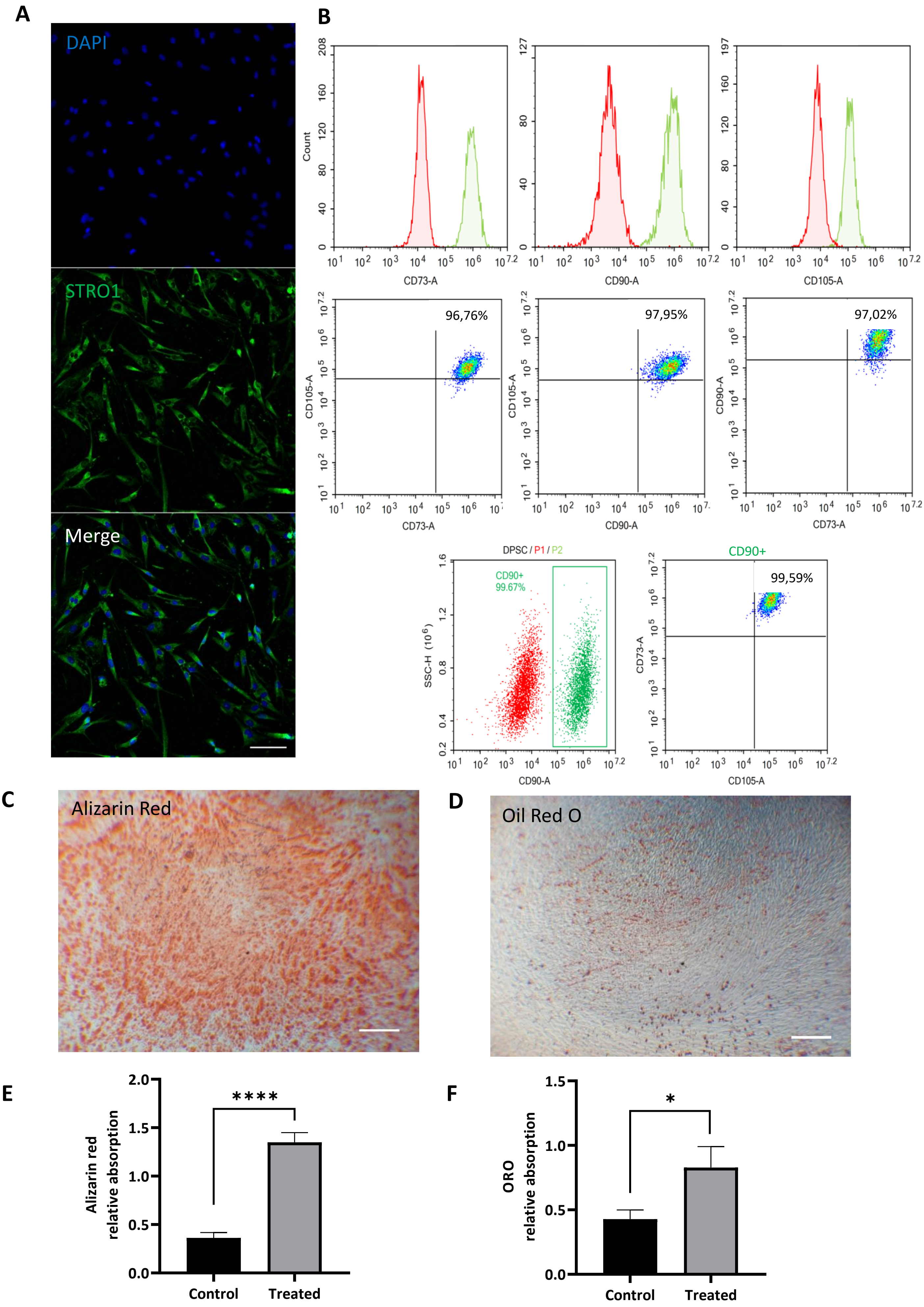

A
DAPI
STRO1
Merge
B
Count
CD73-A
CD90-A
CD105-A
96,76%
97,95%
97,02%
DPSC / P1 / P2
CD90+
99.67%
SSC-H (10^6)
99,59%
C
Alizarin Red
D
Oil Red O
E
****
Alizarin red relative absorption
Control
Treated
F
*
ORO relative absorption
Control
Treated

Figure S1 Identification of mesenchymal stem cell markers and multilineage charaterization of the isolated hDPSCs.

A. hDPSCs from passage 4 were immunostained with an antibody against Stro1 (green). Stro1 immunostaining was detected in virtually all the undifferentiated hDPSCs in culture. Nuclei were stained with DAPI (shown in blue). Scale bar = 100 µm. **B**. Analysis of undifferetiated hDPSCs by flow cytometry indicates the ratio of immunopositive cells for the known mesenchymal characteristic markers: CD73, CD90 and CD105. More than 96% of hDPSCs express either CD73 or CD90 or CD105. Approximatively 99% of CD90+ cells express are CD73 and CD105 positive. **C.** Demonstration of lineage potential of hDPSCs to osteogenic potential after culture in osteogenic medium. The calcium deposit in the matrix was revealed by alizarin red staining. Scale bar = 250 µm. **D.** Demonstration of lineage potential of hDPSCs to adipogenic potential after culture in adipogenic medium. The lipidic vacuoles were revealed by Oil Red O staining. Scale bar = 250 µm. **E.** The calcium deposit in the matrix was quantified by the relative absorbance of alizarin red by spectrophotometry at 545 nm and compared between untreated and treated cultures. The relative absorbance was significantly different between untreated and treated cultures. T-test was used for statistical significance (n=3, ****$P \leq 0.0001$). **F.** Lipidic vacuoles in the cells ware quantified by the relative absorbance of Oil Red O by spectrophotometry at 545 and compared between untreated and treated cultures. The relative absorbance was significantly different between untreated and treated cultures. T test was used for statistical significance (n=3, *$P \leq 0.05$).

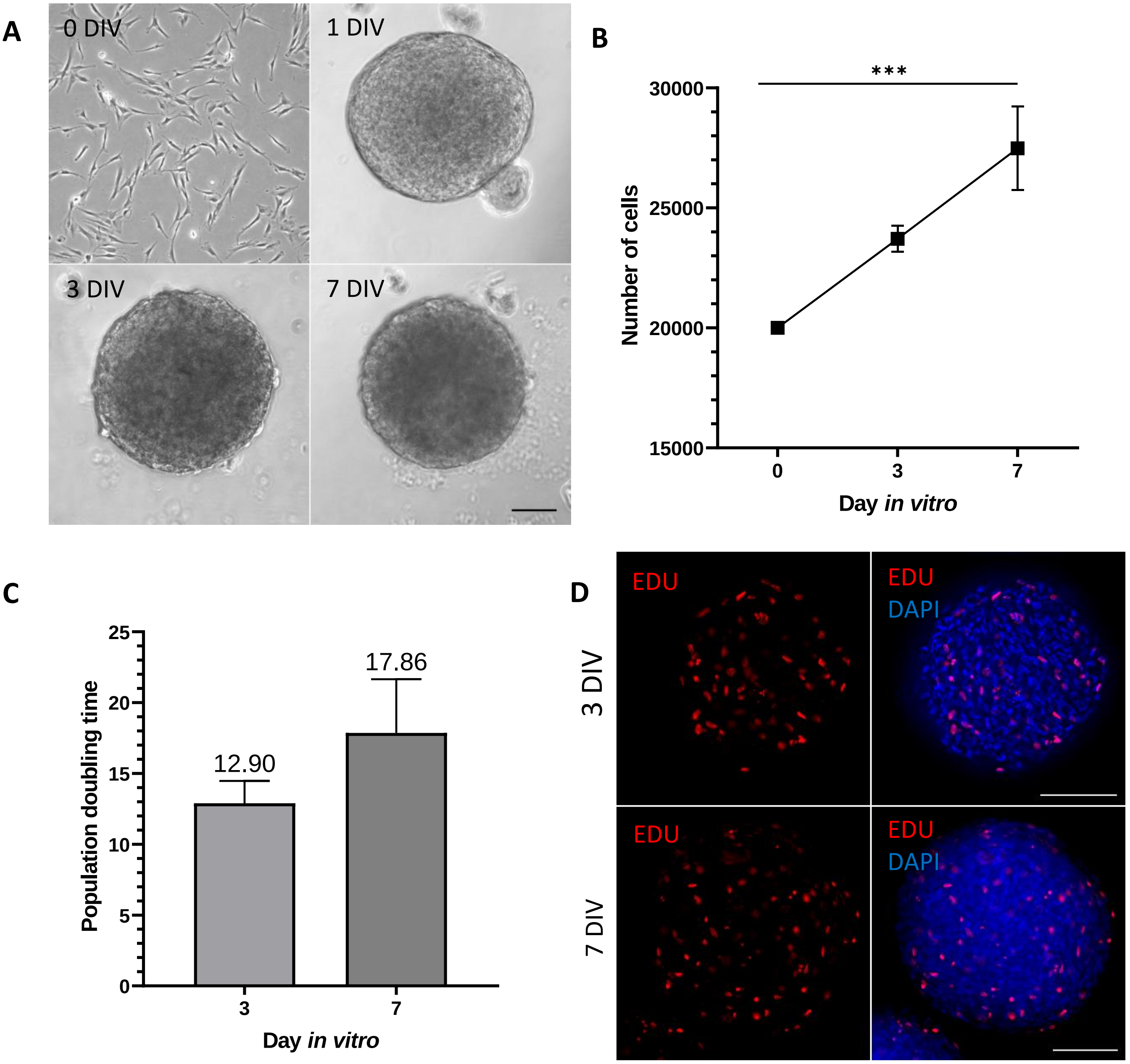

A
0 DIV
1 DIV
3 DIV
7 DIV
B
***
30000
25000
20000
15000
Number of cells
0
3
7
Day *in vitro*
C
25
20
15
10
5
0
Population doubling time
12.90
17.86
3
7
Day *in vitro*
D
EDU
EDU
DAPI
3 DIV
EDU
EDU
DAPI
7 DIV

Figure S2 Morphometric analysis of hDPSC-derived neurospheres.

A. Phase contrast images demonstrate the ability of dissociated hDPSCs to aggregate in floating three-dimensional spheres, as early as 24 h after cell seeding. The generated 3D spheres have semi-transparent appearance. At day 0, cells are in a homogeneous suspension. After 24 h, a 3D sphere is formed by cell aggregation in a medium supplemented with N2 +B27+ bFGF+ EGF. **B**. Quantification of the number of cells per neurosphere. Cells counting demonstrated a progressive increase in cell number at different time points (**day 1 to day 7**) during neurosphere generation to reach ~$27.5 x 10^3$ ± 3000 cells at day 7 *in vitro*. **C**. Evaluation of proliferation ability within the neurospheres by population doubling time (PDT). The cells demonstrated a steadily rising growth with a more evident increase from day 0 up to day 3 *in vitro and* resulting in a PDT of 12.90 (±3.15) and then to a PDT of ~ 17.86 (±7.5) at day 7 *in vitro*. **D**. Assessment of proliferation using Click-it EDU supplied in the culture medium during the period of sphere formation. The EdU is incorporated in the floating spheres with high proliferative capacity. Edu was supplemented in culture medium between day 0 and day 3 then between day 3 and day 7 *in vitro*. Staining indicates that proliferation occurs in both phases of culture. Statistical test T-test was used **$P \leq 0.001$, n=4. Scale bar= 100 µm.

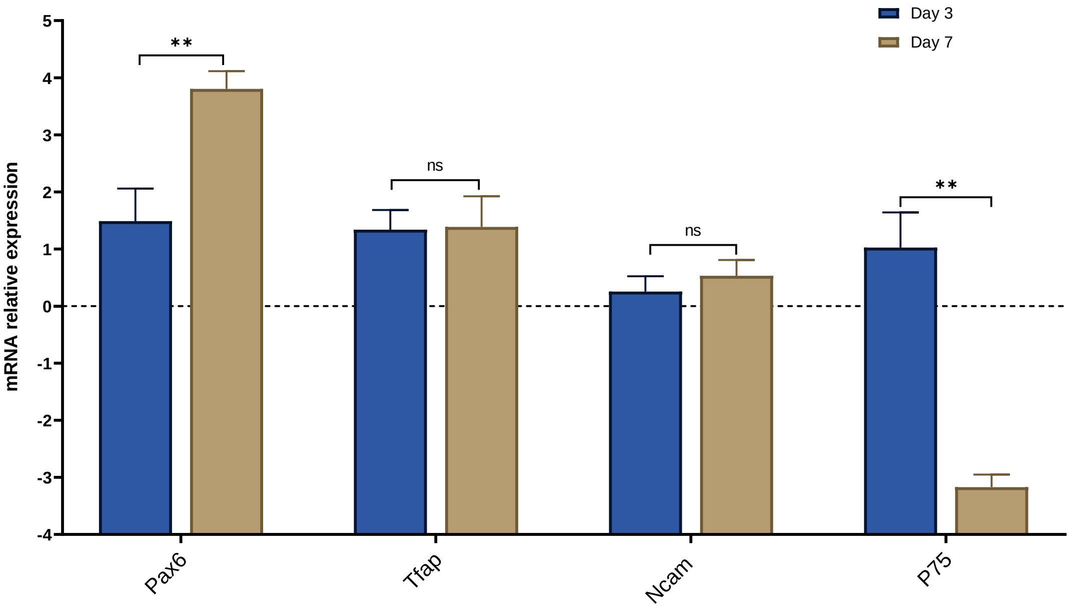

Figure S3 Characterization of Neural progenitors phenotype in neurospheres at 3 & 7 DIV.

Bar charts show the relative gene expression levels in logarithmic (Ln) scale obtained by qPCR analyses for a panel of neural progenitors related lineage. Neurospheres were collected at 3 & 7 DIV. Results are reported to the expression of the genes in undifferentiated cells represented by the dashed line. Bars represent SD. Statistical differences were determined with T-test. P values are indicated with **$P \leq 0.01$, ns = non significant. n= 3.

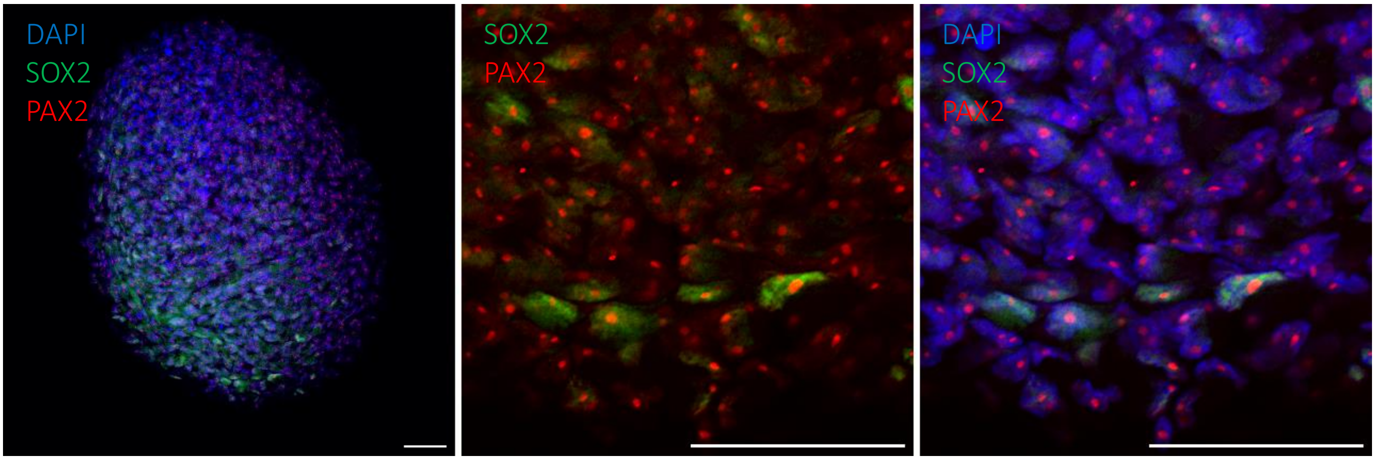

Figure S4 Representative images of immunocytochemical analysis of SOX2 and PAX2 expression in a neurosphere at 7 DIV

Representative images showing SOX2 (shown in green) and PAX2(shown in red) double immunopositive cells at 7 DIV . Cell nuclei were counterstained with DAPI (blue). Scale bars = 50 µm.

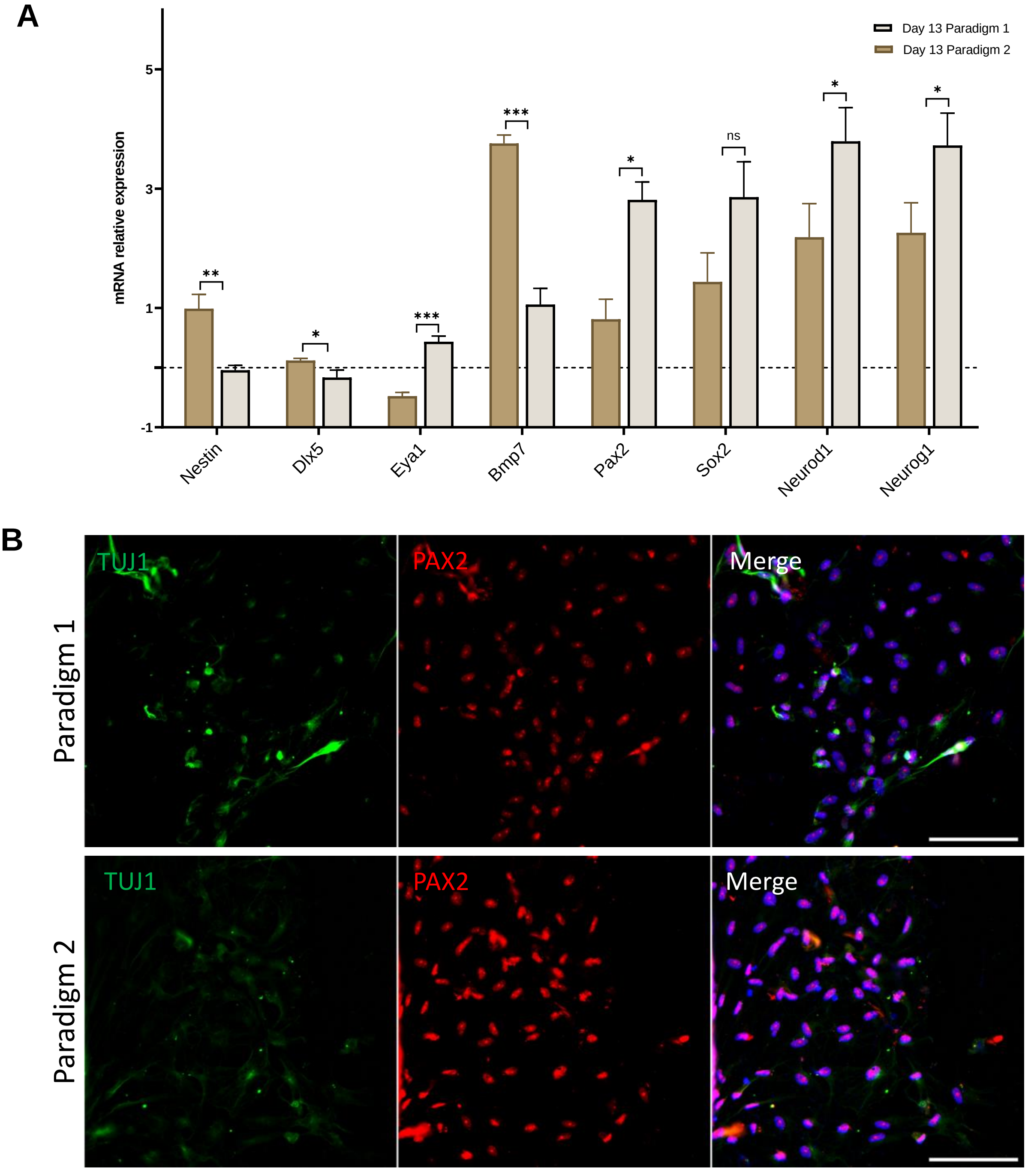

A
Day 13 Paradigm 1
Day 13 Paradigm 2
mRNA relative expression
5
3
1
-1
**
*
***
***
*
ns
*
*
Nestin
Dlx5
Eya1
Bmp7
Pax2
Sox2
Neurod1
Neurog1
B
Paradigm 1
Paradigm 2
TUJ1
PAX2
Merge

Figure S5 Characterization of ONP phenotype in differentiated cells at 13 DIV.

A. Bar charts show the relative gene expression levels in logarithmic (Ln) scale obtained by qPCR analyses for a panel of ONP related lineage from paradigm 1 and from paradigm 2 . Cells were collected at 13 DIV. Results indicate the induction of several genes related to ONP phenotype. Bars represent SD. **B.** Expression of TUJ1 (green), PAX2 (red), PRPH (red) in ONP from paradigms 1 &2 at 13 DIV. Statistical differences were determined with T-test. P values are indicated with *P≤ 0.05, **P≤ 0.01, ***P≤ 0.001. n= 3. Scale bar = 100 µm.

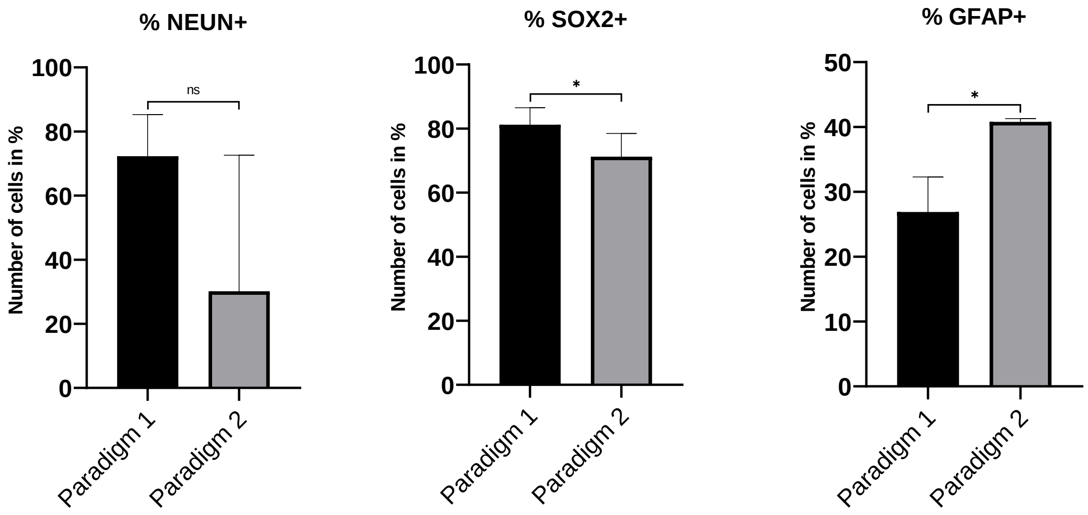

Figure S6 Quantification of the expression of neural markers in cultures at 21 DIV *in vitro* in paradigms 1 and 2

Quantitative analysis revealed a significant number of SOX2 immunopositve cells (about 80 % of total) in paradigm 1 and significant GFAP immunopositive cells (about 40% of total) in paradigm 2 at 21 DIV. Statistical differences were determined with T-test. P values are indicated with *P≤ 0.05, ns= non significant, n=3 different cultures.

A

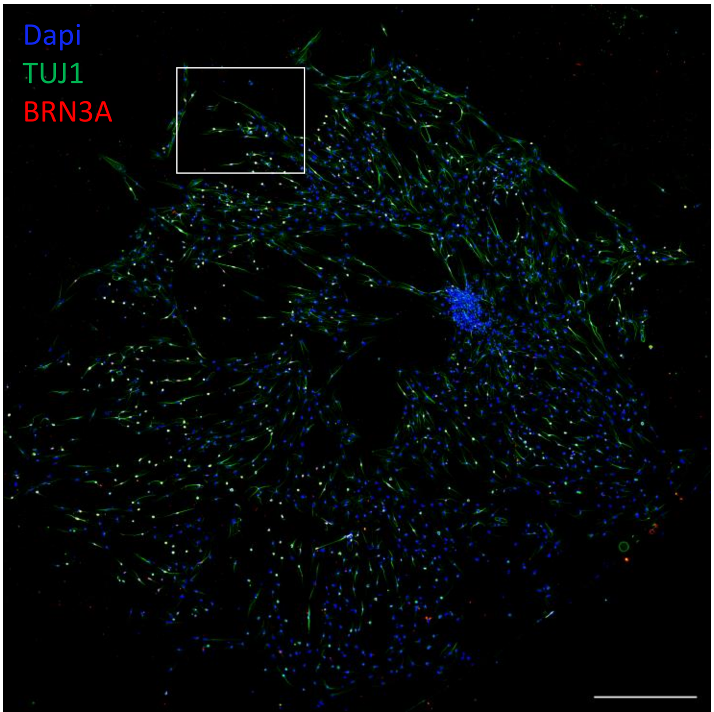

B

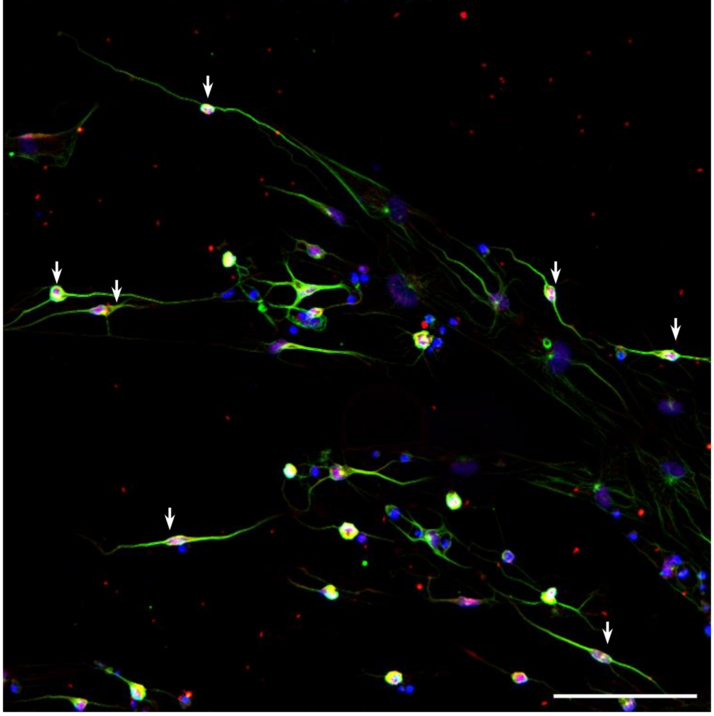

Figure S7 Representative images of immunocytochemical analysis of the expression of BRN3A and TUJ1 at 32 DIV

A. Representative image of SGN like cells at 32 DIV showing TUJ1 (shown in green) and BRN3A(shown in red) . Image show the bipolar morphology of many SGN like cells differentiated at 32 DIV Cell nuclei were counterstained with DAPI (blue). Scale bars = 500 µm.**B.** Magnifiation of the white rectangle in (A) showing bipolar SGN ike cells (arrows) among the diffrentiated neurons expression BRNA3 and TUJ1. Scale bars = 100 µm.

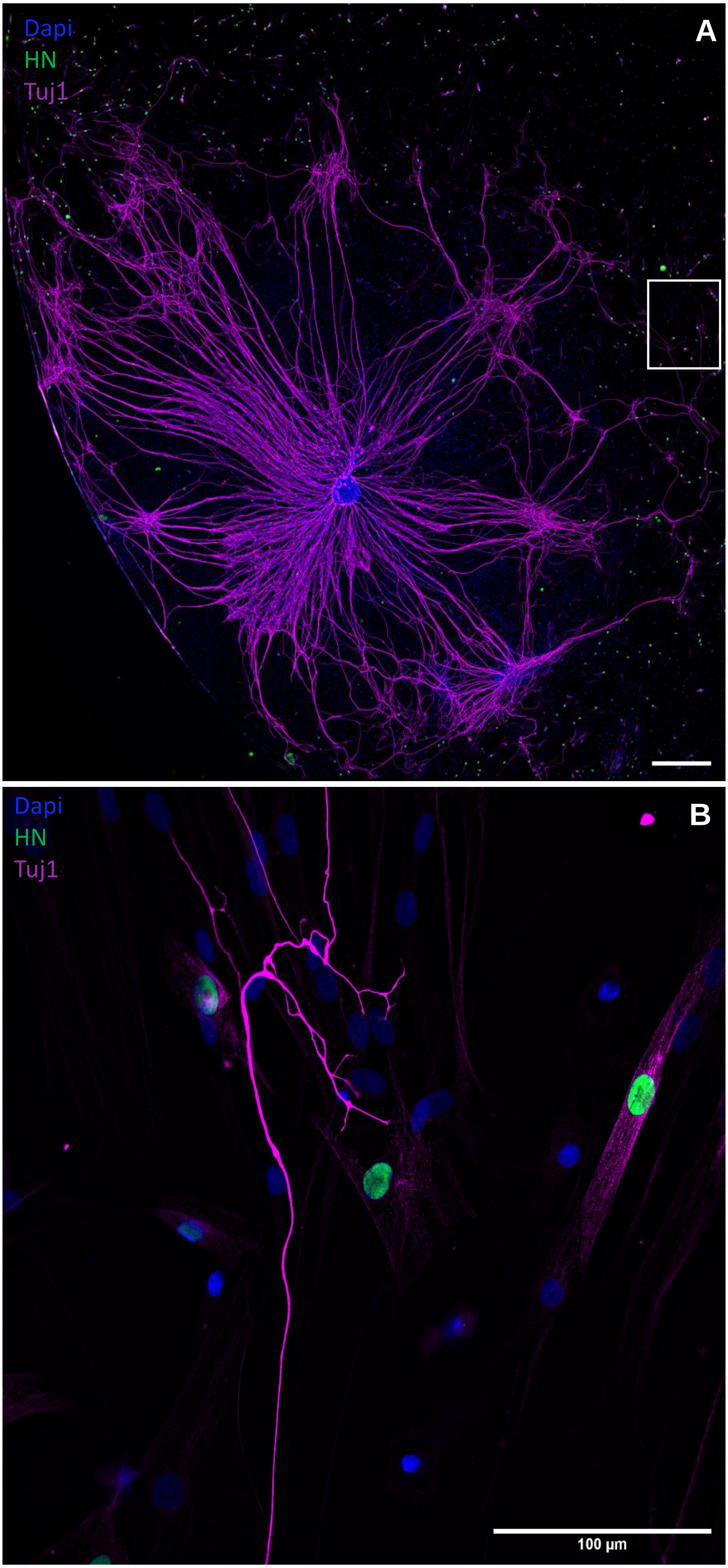
Dapi
HN
Tuj1
A
Dapi
HN
Tuj1
B
100 µm

Figure S8 . Characterization of the co-cultures between human ONPs and rat SG explant.

A. Representative image of neurite outgrowths immunostained with anti-TUJ1 (shown in magenta), projected towards the ONPs immunostained with anti-Human nuclei (shown in green). DAPI was used to counterstain the nuclei. Scale bar = 500 µm. B. Magnification of the area indicated by the white rectangle in (A) showing neurite outgrowths emanating from SG explant (magenta) towards ONP cells (Green). This highlights another observation of contacts between neurites from SG explant and the membrane of ONP cells. Abbreviations, ONPs: otic neuronal progenitors, SG: spiral ganglion.

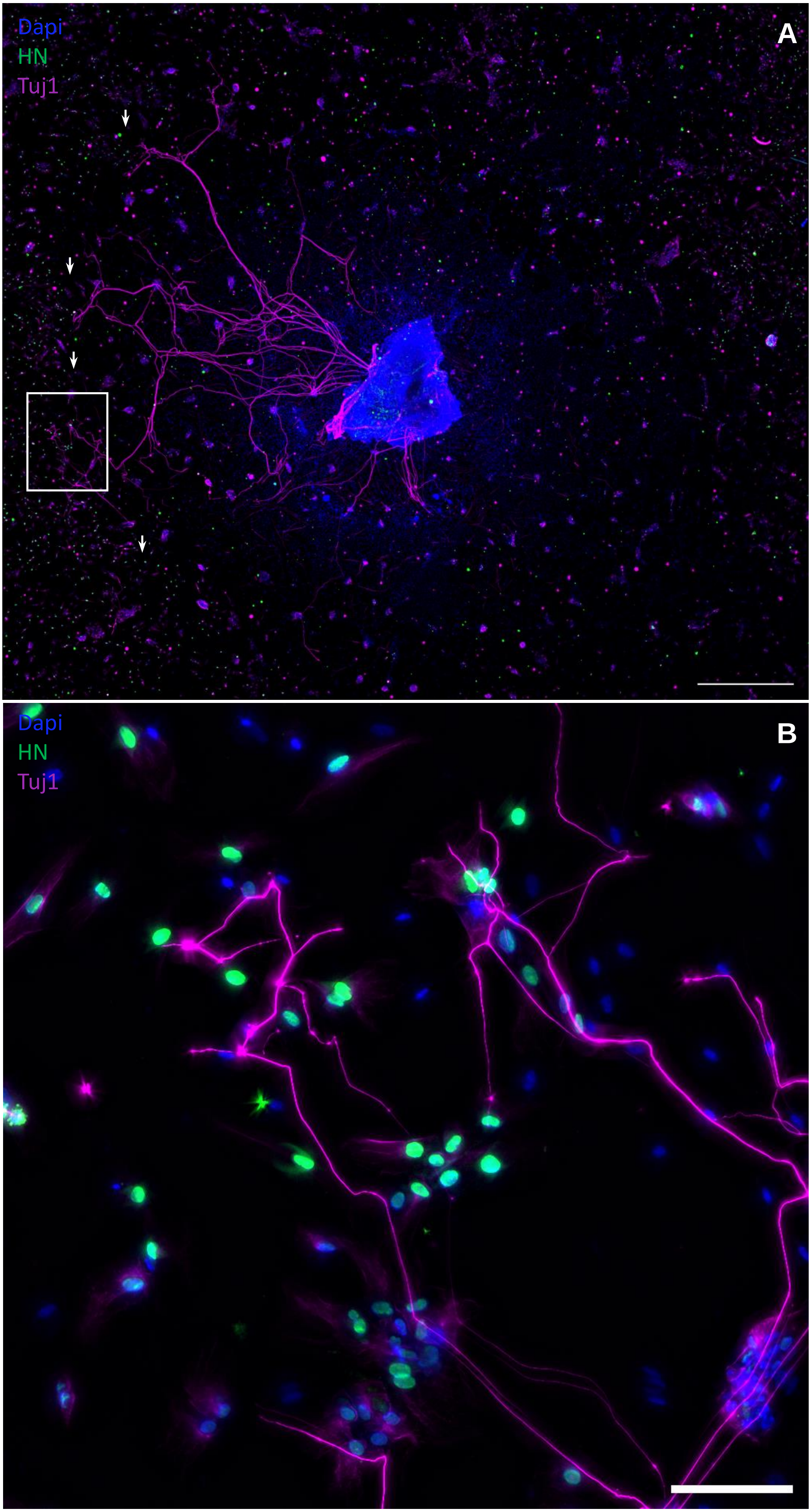
Dapi
HN
Tuj1
A
Dapi
HN
Tuj1
B

Figure S9 . Spiral ganglion neurons from newborn rat send out neurites toward ONPs

A. Representative image of the neurite outgrowths immunostained with anti-TUJ1 (shown in magenta), projected towards the ONPs immunostained with anti-Human nuclei (shown in green). DAPI was used to counterstain the nuclei. Scale bar = 1000 µm. B. Magnification of the area indicated by the white rectangle in (A) showing neurite outgrowths emanating from SG explant (magenta) towards ONP cells (Green). Scale bar = 100 µm. This highlights another observation of contacts between neurites from SG explant and the membrane of ONP cells. Arrows indicate regions where neurites projected towards ONPs . Abbreviations, ONPs: otic neuronal progenitors, SG: spiral ganglion.

List of nonstandard abbreviations

A-MEM	Alpha Modified Eagle Medium
BDNF	Brain-Derived Neurotrophic Factor
BMP	Bone Morphogenic Protein
BMSC	Bone marrow stem cells
DMEM/F12	Dulbecco's Modified Eagle Medium: Nutrient Mixture F-12
DPBS	Dulbecco's Phosphate Buffered Saline
FBS	Fetal Bovine Serum
hDPSC	Human Dental Pulp Stem Cells
HC	Hair Cells
MSC	Mesenchymal Stem Cells
NC	Neural Crest
OC	Organ of Corti
ONP	Otic Neuronal Progenitor
OP	Otic Placode
OV	Otic Vesicle
PBS	Phosphate Buffered Saline
PCR	Polymerase Chain Reaction
P/S	Penicillin Streptomycin
NT-3	Neurotrophin-3
RA	Retinoic Acid
SGN	Spiral Ganglion Neuron
SNHL	Sensorineural Hearing Loss
SV	Stria Vascularis

Table S1. Table of primers

Family	Gene	Forward	Reverse	Access number
Neural Ectoderm	Ncad	TGGGAAATGGAAACTTGATGGC	AATCTGCAGGCTCACTGCTC	NM_001308176.2
	Pax6	TTGCCCGAGAAAGACTAGCA	TCTCCATTTGGCCCTTCGATTA	NM_001368892.2
Non-Neural Ectoderm	Eya1	ACAGCCGACGGGTCTTTAA	TTGGTCGTGGGCTGAAACTA	NM_172058.4
	Six1	GGTTTAAGAACCGGAGGCAAA	TGCTTGTTGGAGGAGGAGTTA	NM_005982.4
	Dlx5	GCTAGCTCCTACCACCAGTAC	GGTTTGCCATTCACCATTCTCA	NM_005221.6
	Tfap	TTTCAGCCATGGACCGTCA	GGGAGATTGACCTACAGTGC	NM_001032280.3
	Foxi1	GACAAGCGCCTCACTCTCA	CCGGCCTTGCTCTTGTTGTA	NM_144769.4
	Pax2	CGGCTGTGTCAGCAAAATCC	GCTTGGAGCCACCGATCA	NM_000278.5
	Pax8	GCCCAGTGTCAGCTCCATTA	GCTGTCCATAGGGAGGTTGAA	NM_013992.4
	Ecad	CAGGAGTCATCAGTGTGGTCA	CAAAATCCAAGCCCGTGGTG	NM_001317184.2
	Lhx2	CAAAAGACGGGCCTCACCAA	CGTAAGAGGTTGCGCCTGAA	XM_006717323.3
	Bmp7	ACGTTCCGGATCAGCGTTTA	CTGTCGAGCAGGAAGAGATCC	NM_001719.3
	Dnmt3a	GAGCGGGTTGTGAGAAGGAA	TCCTGCAATGACCTTGGCTT	NM_153759.3
Neural Crest	Foxd3	CTCATGGCCACCCACCAA	GGAGAGTGGCACGCTAAGAA	NM_012183.3
	Sox10	TCGCGGACCAGTACCC	GCGCTTGTCACTTTCGTTCA	NM_006941.4
	Snail1	CGAGTGGTTCTTCTGCGCTA	GGGCTGCTGGAAGGTAAACT	NM_005985.4
	Sox2	AGCTCGCAGACCTACATGAA	GGAGTGGGAGGAAGAGGTAAC	NM_003106.4
	Plp1	TCCACCCTCAATCCACATTTCC	TGGCTAGTCTGCTTTGTGGC	NM_001128834.3
	Nestin	GTGGCTCCAAGACTTCCCTC	GGTGTCTCAAGGGTAGCAGG	NM_006617.2
	Zic1	GCGCGCTCCGAGAATTTA	CCCTCAAACTCGCACTTGAA	NM_003412.4

	Pax3	GCGGTCTGTGATCGAAACA	TCCTCCTCTTCACCTTTCCC	NM_181461.4
	Pax7	ACAGCATCGACGGCATCC	CAGGTTCCGACTCCACATCC	NM_013945.3
SGN	Ascl1	CGGTCTCATCCTACTCGTCG	CGCCACTGACAAGAAAGCAC	NM_004316.4
	Neurod1	TGACTGATTGCACCAGCCCT	TTCTCAAACTCGGCGGACGG	NM_002500.5
	Neurog1	AGCGCCTTTCTATCTGTCCG	AGGAAGCCGGATAGGTCACT	NM_006161.3
	Neurog2	AGGCCAAAGTCACAGCAACG	CCAAGGTCTCGGATTTGACG	
	Dll1	CTCAGGGGAGGAGAAGGGG	GAGAAACGGGAGTCTTGCCA	NM_005618.4
	Brn3a	CTGAGCACAAGTACCCGTCG	GCTTGAAAGGATGGCTCTTGC	NM_006237.4
	Tlx3	GTTCCAAAACCGGAGGACCA	CTGGATGGAGTCGTTGAGGC	NM_021025.4
	Pou3f4	CCCATTTCGGTTCACCTCCA	GCAGAGAATGCCTATCCCCC	NM_000307.5
	Tbx1	TCGACAAGCTCAAGCTGAC	GCTGGTATCTGTGCATGGAA	NM_080646.2
	Nfl	ACAAGCAGAACGCCGACATC	GGTCTCCTCGCCTTCCAAGA	NM_006158.5
	Nfm	TCCGGCAGTGATCGGAAGAG	AGCCATTTCCCACTTTGTGC	
	Nfh	CCGACATTGCCTCCTACCAG	GCCATCTCCCACTTGGTGTT	NM_021076.4
	Notch1(receptor)	ACGGCGTGAACACCTACAA	TGGCACTCGTCCACATCC	XM_011518717.2
	JAG1 (ligand)	AACAAAGGCTTCACGGGAAC	CAAGTGCCACCGTTTCTACAA	NM_000214.3
	Isl1	TCGCCTTGCAGAGTGACATA	CCCGGTCCTCCTTCTGAAAA	NM_002202.3
	Peripherin	GCCGGAAGACGGTTCTGAT	TAGGGTTTGGGCTTTGAGCA	NM_006262.4
	Trkb	GGAATTGGGTTGGAGCAGGA	GGGGCGCAGATTCCTTGTTA	
	Trkc	CACCCCTTCCTGATGTGGAC	GCCATTGTCCTCACTCGTCA	
	Vglut1	AGGAGCGCAAGTACATCGAG	CGCCAGGGAGTGCTAAACTT	
	Bdnf	AGCCTTTTCCTCCTGCTGTG	GCAGCCTTCATGCAACCAAA	NM_001143805.1
	Nt3	CGCACATCTGGGACCCCT	TGGACATCACCTTGTTCACCT	XM_011520963.2
Schwann Cells	P75	CAGGACAAGCAGAACACCGT	GGTGTGGACCGTGTAATCCA	NM_002507.4
	Ncam	GATGCGACCATCCACCTCAA	TCTCTGGTCGAGTCCACGAA	
	S100	AGGAGCTGAAAGAGCTGCT	TGTCCACAGCATCCACATCC	NM_006271.2

		G		
	Oct6	TGGACTCTTTTTGTTCGGTTG C	CGTCCGGGTGTTTGGTTTTG	NM_002699.4
Endoderm	Cxcr4	CCCGACTTCATCTTTGCCAAC	ACACAACCACCCACAAGTCA	NM_003467.3
	Sox17	CACAACGCCGAGTTGAGCAA	GCTCTGCCTCCTCCACGAA	NM_022454.4
	Gata4	AAAACGGAAGCCCAAGAAC C	AAGGCTCTCACTGCCTGAA	NM_001308094 .2
	Gata6	GGGCTCTACAGCAAGATGA AC	GTTGGCACAGGACAATCCAA	NM_005257.6
	Epcam	GTGCTGGTGTGTGAACACTG	GAAGTGCAGTCCGCAAACTT	NM_002354.3
	FoxA2	ACTGGAGCAGCTACTATGCA	TGTTCATGCCGTTCATCCC	NM_021784.5
Mesoderm	Bra/T	CGCTTCAAGGAGCTCACCAA	GCCAGACACGTTCACCTTCA	NM_003181.4
	Hand1	CAAGCGGAAAAGGGAGCTG	CAGCCGGTGCGTCCTTTAAT	
	Sox7	GGCCAAGGACGAGAGGAAA C	TCCGCCTCGTCCACGTA	NM_031439.4
	Mixl1	GTACCCCGACATCCACTTGC	ACCTGGAAGAGGGGAGAAAA TAA	
Housekeeping	Rps18	CCGCCATGTCTCTAGTGATC C	GGTGAGGTCGATGTCTGCTT	NM_011296.2

Table S2. Table of primary antibodies

Antigen	Host	Brand	Ref	Dilution
SOX2	Mouse	Abcam	ab97959	1:100
SOX2	Rabbit	Merck	AB5603	1:200
STRO1	Mouse	Invitrogen	398401	1:150
NESTIN	Mouse	Abcam	ab18102	1:250
PAX2	Rabbit	Abcam	ab79389	1:200
BRN3a	Rabbit	Sigma Aldrich	AB5945	1:150
Human nuclear AG	Mouse	Abcam	ab191181	1:200
B3-TUBULIN	Mouse	Abcam	ab78078	1:250
B3-TUBULIN	Rabbit	Abcam	ab18207	1:500
MAP2	Mouse	Invitrogen	13-1500	1:200
TRKc	Rabbit	Cell signaling	C44H5	1:100
NEUN	Rabbit	Cell signaling	D3S3I	1:100
PERIPHERIN	Rabbit	Abcam	ab4666	1:250
NF-M	Mouse	DSHB	2H3-C	1:200
GFAP	Mouse	DSHB	8-1E7-S	1:150
